# Supplementary material for: Finger sweat analysis enables short interval metabolic biomonitoring in humans
Source: Nat Commun. 2021 Oct 13;12:5993. doi: 10.1038/s41467-021-26245-4 (PMC8514494; doi:10.1038/s41467-021-26245-4)
Supplement: Supplementary file 1 — Supplementary Information [file 41467_2021_26245_MOESM1_ESM.pdf]

Supplementary Information to

Finger Sweat Analysis Enables High-Frequency Biomonitoring in Humans

Julia Brunmair[†1], Mathias Gotsmy[†1], Laura Niederstaetter[1], Benjamin Neuditschko[1,2], Andrea Bileck[1,3], Astrid Slany[1], Max Lennart Feuerstein[1], Clemens Langbauer[1], Lukas Janker[1,3], Jürgen Zanghellini[1], Samuel M. Meier-Menches[1–3], and Christopher Gerner[1,3]

[1]Department of Analytical Chemistry, Faculty of Chemistry, University Vienna
[2]Department of Inorganic Chemistry, Faculty of Chemistry, University Vienna
[3]Joint Metabolome Facility, Faculty of Chemistry, University Vienna

[†]Both authors contributed equally

Contents

1 Supplementary Tables 3

2 Supplementary Figures 15

3 Supplementary Note 1: Caffeine metabolisation in HepG2 cells 30

3.1 Cell culture conditions . . . 30
3.2 Metabolite Extraction . . . 30

4 Supplementary Note 2: Targeted approach using multiple reaction monitoring (MRM) 31

4.1 Chemicals . . . 31
4.2 Standard Solutions and Calibration Samples . . . 31
4.3 Samples . . . 31
4.4 Sample Preparation . . . 31
4.5 Targeted LC-MS/MS . . . 31
4.6 Mass Spectrometry . . . 32

5 Supplementary Note 3: Mathematical model 33

6 Supplementary Note 4: Sensitivity analysis 36

6.1 Aim . . . 36
6.2 Methods . . . 36
6.3 Results . . . 38
6.4 Discussion . . . 40

7 Supplementary References 41

1 Supplementary Tables

Supplementary Table 1: Caffeine metabolites detected in finger sweat and verified with external analytical standards. n.d. = not detected.

**Compound**	**Chemical Formula**	**Monoisotopic Mass**	**pos Precursor [M+H]+**	**neg Precursor [M-H]-**	**mass error [ppm]**	**RT [min]**
1.3.7-Trimethyluric acid	C8H10N4O3	210.0753	n.d.	209.0680	0.96	2.86
1.7-Dimethyluric acid	C7H8N4O3	196.0596	n.d.	195.0524	0.00	2.44
1-Methyluric Acid	C6H6N4O3	182.0440	n.d.	n.d.	n.d.	n.d.
1-Methylxanthine	C6H6N4O2	166.0491	167.0564	165.0413	0.60	1.75
3.7-Dimethyluric acid	C7H8N4O3	196.0596	n.d.	195.0524	0.00	2.06
3-Methyluric acid	C6H6N4O3	182.0440	n.d.	181.0367	0.55	1.52
3-Methylxanthine	C6H6N4O2	166.0491	167.0564	165.0413	0.00	1.60
5-Acetylamino-6-formyl- amino-3-methyluracil	C8H10N4O4	226.0702	227.0775	n.d.	0.44	1.09
7-Methyluric acid	C6H6N4O3	182.0440	n.d.	181.0367	0.55	1.34
7-Methylxanthine	C6H6N4O2	166.0491	167.0564	165.0413	1.79	1.48
Caffeine	C8H10N4O2	194.0804	195.0877	n.d.	0.51	3.28
Paraxanthine	C7H8N4O2	180.0647	181.0720	n.d.	0.00	2.55
Theobromine	C7H8N4O2	180.0647	181.0720	n.d.	0.55	2.14
Theophylline	C7H8N4O2	180.0647	181.0720	n.d.	1.10	2.65
Uric Acid	C5H4N4O3	168.0283	169.0356	167.0205	1.18	0.78
Xanthine	C5H4N4O2	152.0334	153.0407	151.0256	1.31	0.95

Supplementary Table 2: Estimated parameters. Kinetic parameters [h^{-1}], initial (unitless) concentrations and sweat volumes (V_{sweat}) [µL] fitted by the kinetic model (Figure 5a) with their respective 95% confidence intervals (calculated from CVs from Supplementary Table 15, n = 120, df = 93) for profile 1 and profile 2. Moreover, the median of kinetic constants of the whole sampled cohort and their relative standard deviation are shown in the population estimate column. As comparison the kinetic parameters found in blood plasma after uptake of 5 mg caffeine per kg specimen body mass are listed [1].

**Parameter**	**Profile 1**	**Profile 2**	**Population estimate**	**Bonati et al.** [1]
k_1	5.620 h^{-1} ± 57%	4.584 h^{-1} ± 57%	4.404 h^{-1} ± 72%	$(6.31 \pm 1.91)h^{-1}$
k_2	0.036 h^{-1} ± 36%	0.025 h^{-1} ± 36%	0.036 h^{-1} ± 64%	$(0.099 \pm 0.022)h^{-1}$
k_3	0.016 h^{-1} ± 35%	0.110 h^{-1} ± 35%	0.008 h^{-1} ± 228%	$(0.015 \pm 0.002)h^{-1}$
k_4	0.006 h^{-1} ± 35%	0.003 h^{-1} ± 35%	0.004 h^{-1} ± 58%	$(0.013 \pm 0.001)h^{-1}$
k_5	0.001 h^{-1} ± 74%	0.000 h^{-1} ± 74%	0.010 h^{-1} ± 207%	-
k_6	0.137 h^{-1} ± 35%	0.081 h^{-1} ± 35%	0.141 h^{-1} ± 42%	-
k_7	0.116 h^{-1} ± 40%	0.114 h^{-1} ± 40%	0.048 h^{-1} ± 108%	-
k_8	0.119 h^{-1} ± 40%	0.061 h^{-1} ± 40%	0.072 h^{-1} ± 65%	-
c_0^{par}	0.026 ± 38%	0.008 ± 38%	-	-
c_0^{bro}	0.028 ± 37%	0.223 ± 37%	-	-
c_0^{phy}	0.006 ± 38%	0.004 ± 38%	-	-
$V_{\text{sweat}}(0)$	0.62 µL ± 40%	2.31 µL ± 40%	-	-
$V_{\text{sweat}}(15)$	2.60 µL ± 49%	1.94 µL ± 49%	-	-
$V_{\text{sweat}}(30)$	1.36 µL ± 36%	1.02 µL ± 36%	-	-
$V_{\text{sweat}}(45)$	1.42 µL ± 38%	0.79 µL ± 38%	-	-
$V_{\text{sweat}}(60)$	1.72 µL ± 45%	0.54 µL ± 45%	-	-
$V_{\text{sweat}}(90)$	1.30 µL ± 31%	0.53 µL ± 31%	-	-
$V_{\text{sweat}}(120)$	1.53 µL ± 25%	0.13 µL ± 25%	-	-
$V_{\text{sweat}}(180)$	1.51 µL ± 31%	0.44 µL ± 31%	-	-
$V_{\text{sweat}}(240)$	1.70 µL ± 27%	0.92 µL ± 27%	-	-
$V_{\text{sweat}}(360)$	1.77 µL ± 29%	0.59 µL ± 29%	-	-
$V_{\text{sweat}}(480)$	2.35 µL ± 33%	1.44 µL ± 33%	-	-
$V_{\text{sweat}}(1440)$	1.84 µL ± 65%	2.44 µL ± 65%	-	-
$V_{\text{sweat}}(1500)$	1.14 µL ± 76%	3.19 µL ± 76%	-	-
$V_{\text{sweat}}(1460)$	1.35 µL ± 73%	1.44 µL ± 73%	-	-
$V_{\text{sweat}}(1620)$	4.00 µL ± 72%	2.53 µL ± 72%	-	-

Supplementary Table 3: Fractional conversion of caffeine to degradation products. The fractional conversions of caffeine to degradation products (paraxanthine, theobromine, theophylline) in % for profile 1 and 2 and a comparison to literature are given. Values from profile 1, 2 and Bonati et al. were calculated with Equation 17.

**Degradation Product**	**Profile 1**	**Profile 2**	**Bonati et al. [1]**	**Lelo et al. [2]**
Paraxanthine	66	12	78 ± 17	84 ± 5
Theobromine	22	86	12 ± 1	12 ± 4
Theophylline	13	2	10 ± 2	4 ± 1

Supplementary Table 4: Parameters for the targeted LC-MS/MS approach.

**Analyte**	**Precursor Ion**	**Product Ion**	**Dwell-time [ms]**	**Fragmentor/Collision Energy [eV]**
Caffeine	195.1	138	50	380/40 (quantifier)
		110	50	380/40
		83	50	380/40
Theophylline/ Paraxanthine	181	123.9	50	380/30
		95.9	50	380/30
		69	50	380/30 (quantifier)
Theobromine	181	122.2	50	380/30
		107.9	50	380/30
		67	50	380/30 (quantifier)

Supplementary Table 5: Analytical validation of CF and its metabolites in the proof-of-principle study. The compounds were spiked in two concentrations. The precision for CF, TB and TP is between 0.03 and 15.6% with overall process efficiency from 88—92%, including the associated coefficients of variation (CV) in brackets. The LLOQ for caffeine and its metabolites was defined as the lowest concentration giving signal-to-noise ratio of at least 10:1. The lowest concentration that can be detected, with a signal-to-noise ratio of 3:1, is specified as the limit of detection (LOD).

**Compound**	**Spiked conc. [pg µL-1]**	**Precision [%]**	**Overall Process Efficiency (CV)**	**LLOQ [pg/FP]**	**LOD [pg/FP]**
Caffeine	0.25	15.9	-	0.27	0.11
	25	0.03	88.4 (7.2%)		
Theobromine	0.25	4.1	-	0.34	0.14
	25	0.7	92.0 (5.9%)		
Theophylline	0.25	1.9	-	0.21	0.10
	25	0.2	89.9 (7.5%)		

Supplementary Table 6: List of symbols used throughout the manuscript.

Symbol	Name	Unit
free_caf	external caffeine	
caf	internal caffeine	
par	internal paraxanthine	
bro	internal theobromine	
phy	internal theophylline	
f	bioavailability	%
M_{donor}	donor body mass	kg
M_{dose}	mass of ingested external caffeine	g
$\tilde{M}$	measured mass of metabolites	g
$\tilde{m}$	specific measured mass of metabolites	L
V_{D}	volume of distribution	L
v_{D}	specific volume of distribution	$\text{L}\,\text{kg}^{-1}$
V_{sample}	sample volume	L
V_{sweat}	sweat volume	L
C	theoretical concentration of metabolites	$\text{g}\,\text{L}^{-1}$
c	specific theoretical concentration of metabolites	
$\tilde{C}$	measured concentration of metabolites	$\text{g}\,\text{L}^{-1}$
$\tilde{c}$	specific measured concentration of metabolites	
a	slope of the calibration curve	$\text{g}\,\text{L}^{-1}\,\text{nAUC}^{-1}$
fc	fractional conversion	
$k_1 - k_9$	kinetic parameters of the model	h^{-1}
t	time	h
τ	set of time points	
j	number of time points	
i	number of bootstrap replicates of the simulation	
n	number of Monte Carlo replicates of the fit	
$\mu_{V_{\text{sweat}}}$	center of the simulated V_{sweat} distribution	L
$\sigma_{V_{\text{sweat}}}$	standard deviation of the simulated V_{sweat} distribution	L
μ_{ϵ}	center of the simulated error distribution	
σ_{ϵ}	standard deviation of the simulated error distribution	
CV	coefficient of variation	%
MRE	median of the relative error	%

Supplementary Table 7: List of constants and fixed parameters.

$v_{\mathrm{D}}^{\mathrm{caf}}/f$	$0.579\,\mathrm{L\,kg^{-1}}$ [3]	specific volume of distribution divided by bioavailability
M_{dose}	$200\,\mathrm{mg}$	dose
V_{sample}	$123\,\mathrm{\mu L}$	sample volume

Supplementary Table 8: Measured constants of the calibration curve. The calibration curve is of the $y^i = a^i x^i$, $i \in \{\mathrm{caf, par, bro, phy}\}$.

metabolite	caf	par	bro	phy
a^i / $[\mathrm{pg\,\mu L^{-1}\,nAUC^{-1}}]$	1.520	1.656	2.050	1.592

Supplementary Table 9: Boundaries of the model parameters and values used in the simulations.

	lower bound	upper bound	simulation values	unit
k_1	0	10	1.60	$\mathrm{h^{-1}}$
k_2	0	0.20	0.02	$\mathrm{h^{-1}}$
k_3	0	0.20	0.01	$\mathrm{h^{-1}}$
k_4	0	0.20	0.01	$\mathrm{h^{-1}}$
k_5	0	0.20	0.04	$\mathrm{h^{-1}}$
k_6	0	0.20	0.13	$\mathrm{h^{-1}}$
k_7	0	0.20	0.10	$\mathrm{h^{-1}}$
k_8	0	0.20	0.10	$\mathrm{h^{-1}}$
c_0^{par}	0	0.20	0.02	
c_0^{bro}	0	0.20	0.01	
c_0^{phy}	0	0.20	0.01	
V_{sweat}	0.05	4	$\mathcal{N}(1.34, 0.57^2)$	$\mathrm{\mu L}$

Supplementary Table 10: Coefficients of variation (CV in %) and the median of relative errors (MRE in %) of kinetic parameters, the sweat volume and concentrations are given for different loss functions. The word 'Max' in the loss row indicates that the loss was calculated from the maximum of absolute or relative errors. Otherwise only absolute error values were used. For all simulations the number of inner Monte Carlo replicates, n, was 100 and the number of outer MC replicates, i, was 300, and the number of time points, j, was 20.

Loss	Cauchy		Huber		Soft-l1		Robust		Max Cauchy		Max Huber		Max Soft-l1		Max Robust	
	CV	MRE	CV	MRE	CV	MRE	CV	MRE	CV	MRE	CV	MRE	CV	MRE	CV	MRE
k_1	39.19	-2.85	35.8	2.38	35.15	-2.09	36.75	7.18	29.79	0.89	30.01	1.04	32.49	32.15	25.56	1.48
k_2	25.48	2.6	23.48	2.38	23.85	2.41	16.7	2.64	18.2	0.28	17.76	1.31	16.81	16.79	19.08	4.07
k_3	24.52	3.53	23.1	4.15	22.71	2.91	23.93	9.86	15.94	-0.45	16.12	0.21	15.61	15.58	19.08	4.19
k_4	24.81	2.32	22.62	1.68	23.13	0.44	24.18	11.55	16.89	-0.54	15.76	1.52	15.65	15.6	18.44	2.24
k_5	81.55	71.73	70.77	53.8	78.82	67.36	55.19	24.54	48.68	-32.01	78.73	-83.26	78.03	25.35	41.22	-13.41
k_6	35.6	30.6	33.33	23.21	34.78	28.52	22.88	12.39	24.28	-10.66	32.79	-21.63	32.75	24.33	22.63	0.92
k_7	42.88	34.44	41.69	30.39	41.75	33.24	40.96	26.99	29.37	-13.3	39.11	-29.94	38.58	27.21	28.69	3.32
k_8	42.61	33.9	41.32	27.8	40.9	32.07	42.11	30.34	29.77	-13.37	37.78	-27.81	38.32	26.79	26.86	2.84
c_0^{par}	25.99	-1.57	24.43	-1.18	24.18	-2.09	21.63	-0.41	17.44	-0.35	18.22	-1.78	18.26	18.19	17.64	-0.73
c_0^{bro}	25.16	-1.8	24.96	-1.36	23.46	-2.3	35.14	-6.47	16.68	-1.71	18.02	-2.16	17.35	17.24	18.17	0.48
c_0^{phy}	25.52	-0.82	24.48	0.09	24.11	-1.12	35.92	-9.76	17.15	-0.23	17.16	-3.43	16.86	16.74	18.11	-0.13
V_{sweat}	45.93	16.42	41.79	12.77	43.97	15.58	33.29	5.96	24.09	-7.15	26.93	-14.35	23.64	19.05	19.66	-2.57
c_{caf}	22.55	-14.23	20.49	-10.88	21.4	-13.17	16.08	-5.22	17.8	7.01	28.68	16.48	28.5	13.32	12.88	1.51
c_{par}	23.06	-14.56	21.06	-11.48	22.05	-13.85	16.98	-5.8	18.22	7.2	29.58	16.62	29.66	14.76	14.49	2.91
c_{bro}	22.85	-14.77	21.18	-11.85	22.1	-14.08	19.46	-6.54	18.31	6.85	29.46	16.54	28.94	14.5	14.48	3.2
c_{phy}	22.75	-14.35	21.1	-11.41	22.23	-14.3	19.77	-6.68	18.33	6.95	29.48	16.43	29.12	14.49	14.61	2.89
average	33.15	16.28	30.72	12.93	31.54	15.35	28.81	10.77	22.56	6.81	29.1	15.91	28.79	19.51	20.72	2.93

Supplementary Table 11: Coefficients of variation (CV in %) and the median of relative errors (MRE in %) of kinetic parameters, the sweat volume and concentrations are given for different numbers of Monte Carlo replicates (n). For all simulations i was 300, j was 20 and the max Robust loss was used.

n	1		10		100		1000	
	CV	MRE	CV	MRE	CV	MRE	CV	MRE
k_1	41.73	-3.69	28.39	-2.81	25.56	1.48	24.39	2.53
k_2	19.72	3.96	19.31	6.8	19.08	4.07	16.93	1.11
k_3	23.12	7.07	19.7	5.38	19.08	4.19	15.95	3.01
k_4	23.52	5.93	20.37	6.86	18.44	2.24	15.34	1.98
k_5	74.63	58.1	46.84	21.82	41.22	-13.41	44.01	-33.52
k_6	31.2	24.13	26.68	14.12	22.63	0.92	22.3	-7.36
k_7	43.39	31.37	33.05	19.17	28.69	3.32	25.72	-7.53
k_8	44.28	31.3	32.75	19.65	26.86	2.84	24.48	-6.68
c_0^{par}	20.69	-0.7	19.42	-2.44	17.64	-0.73	17.94	2.26
c_0^{bro}	21.18	-1.04	19.08	-2.23	18.17	0.48	16.73	2.05
c_0^{phy}	21.06	-1.82	19.34	-1.86	18.11	-0.13	16.43	1.22
V_{sweat}	39.81	12.93	26.83	5.2	19.66	-2.57	18.27	-6.98
c^{caf}	20.69	-12.75	14.53	-5.6	12.88	1.51	14.54	5.5
c^{par}	19.93	-11.73	15.27	-4.88	14.49	2.91	16.83	7.25
c^{bro}	19.86	-11.45	15.04	-4.9	14.48	3.2	16.59	7.3
c^{phy}	19.9	-11.47	15.19	-4.39	14.61	2.89	16.72	7.35
average	30.29	14.34	23.24	8.01	20.72	2.93	20.2	6.48

Supplementary Table 12: Number (j) and values (τ) of time points (in h) investigated. During the caffeine capsule study we improved the sampling intervals. Therefore, we did simulations resembling the **e**arlier sampling intervals (simulation id starting with an E, for donor 1–15) as well as **l**ater sampling intervals (id starting with an L, for donor 16–47).

id	j	τ / [h]
L11	11	0, 0.25, 0.5, 0.75, 1, 1.5, 2, 3, 4, 6, 8
L13	13	0, 0.25, 0.5, 0.75, 1, 1.5, 2, 3, 4, 5, 6, 7, 8
L15	15	0, 0.25, 0.5, 0.75, 1, 1.5, 2, 3, 4, 5, 6, 7, 8, 9, 10
L17	17	0, 0.25, 0.5, 0.75, 1, 1.5, 2, 3, 4, 5, 6, 7, 8, 9, 10, 11, 12
L19	19	0, 0.25, 0.5, 0.75, 1, 1.5, 2, 3, 4, 5, 6, 7, 8, 9, 10, 11, 12, 13, 14
L20	20	0, 0.25, 0.5, 0.75, 1, 1.5, 2, 3, 4, 5, 6, 7, 8, 9, 10, 11, 12, 13, 14, 24
E15	15	0, 0.25, 0.5, 0.75, 1, 1.5, 2, 3, 4, 6, 8, 24, 25, 26, 27

Supplementary Table 13: CV (in %) and MRE (in %) calculated for simulations with different numbers of time points (j). Sample times are listed in Table 14. For all simulations i was 300, n was 100 and the max Robust loss was used.

j	20		19		17		15		13		11		$15^{\dagger}$	
	CV	MRE	CV	MRE	CV	MRE	CV	MRE	CV	MRE	CV	MRE	CV	MRE
k_1	25.56	1.48	27.23	7.67	28.47	8.23	27.0	8.06	30.2	14.19	31.45	14.87	27.83	6.0
k_2	19.08	4.07	21.87	-0.15	23.35	-1.66	24.41	-3.92	25.59	-4.17	27.47	-9.14	18.07	-1.38
k_3	19.08	4.19	22.28	2.14	24.45	1.23	23.76	4.07	25.82	-2.93	25.64	-2.38	17.88	1.3
k_4	18.44	2.24	22.08	2.88	23.72	0.85	24.4	3.24	25.86	-0.49	25.45	-5.84	18.15	-0.06
k_5	41.22	-13.41	44.62	-4.35	47.88	-0.31	52.09	-0.82	69.44	8.34	85.06	27.27	36.21	-9.45
k_6	22.63	0.92	29.64	1.46	35.86	0.46	41.07	-3.28	45.62	-3.17	51.49	-10.17	16.55	-0.85
k_7	28.69	3.32	37.39	4.09	43.01	-3.13	48.93	9.43	60.3	-3.87	59.36	7.12	19.57	-0.76
k_8	26.86	2.84	37.69	5.17	44.38	1.53	50.7	9.69	61.86	7.91	60.0	-5.73	19.53	-0.24
c_0^{par}	17.64	-0.73	19.48	2.98	19.69	6.57	18.25	6.41	19.07	8.03	19.55	10.15	18.5	5.1
c_0^{bro}	18.17	0.48	18.4	2.51	19.26	2.93	17.24	4.55	19.52	7.68	19.2	10.9	18.23	3.88
c_0^{phy}	18.11	-0.13	18.91	4.71	18.55	4.24	17.59	5.31	19.01	6.95	19.45	10.24	18.15	4.07
V_{sweat}	19.66	-2.57	26.06	-1.46	16.4	-2.46	18.83	-2.25	20.0	-2.41	18.55	-2.65	21.56	-3.36
c^{caf}	12.88	1.51	11.61	0.08	11.23	0.96	10.41	0.76	10.84	1.14	11.91	0.65	16.8	1.5
c^{par}	14.49	2.91	13.67	1.42	13.41	2.53	12.62	2.48	13.1	2.29	13.64	2.52	18.67	3.31
c^{bro}	14.48	3.2	13.52	1.47	13.63	2.4	12.6	2.58	13.33	2.84	13.85	3.47	18.75	3.45
c^{phy}	14.61	2.89	13.34	2.16	13.49	3.05	12.61	2.51	13.14	2.47	13.99	2.99	18.62	3.27
average	20.72	2.93	23.61	2.79	24.8	2.66	25.78	4.34	29.54	4.93	31.0	7.88	20.19	3.0

Supplementary Table 14: CV (in %) and MRE (in %) calculated for time-dependent parameters at different time points (t). The simulation was done with $n = 100$, $i = 3000$, $j = 20$ (L20), and max Robust loss. Since the model forces $c_0^{\text{caf}} = 0$ no CV and MRE were calculated for this instance.

t	V_{sweat}		c^{caf}		c^{par}		c^{bro}		c^{phy}	
	CV	MRE	CV	MRE	CV	MRE	CV	MRE	CV	MRE
0	23.63	-0.69	-	-	17.12	1.07	16.95	0.45	17.02	0.56
0.25	21.63	-2.51	19.63	1.44	16.97	1.14	16.77	0.51	16.83	0.80
0.5	22.30	-2.35	15.33	1.22	16.00	1.58	15.80	0.89	15.83	1.39
0.75	23.24	-2.41	11.99	1.07	14.55	1.92	14.38	1.70	14.42	1.82
1	20.82	-2.20	9.37	0.88	13.00	2.22	12.87	2.16	12.91	2.29
1.5	16.62	-1.34	5.76	0.43	10.29	2.41	10.24	2.45	10.33	2.39
2	14.13	-1.68	3.77	0.01	8.47	2.24	8.51	2.42	8.65	2.20
3	11.67	-1.49	3.36	-0.01	7.22	2.13	7.36	2.38	7.57	2.29
4	19.23	-2.19	4.67	0.44	7.62	2.35	7.80	2.48	8.03	2.42
5	20.86	-1.92	6.09	0.81	8.49	2.48	8.70	2.89	8.90	2.51
6	14.13	-2.72	7.48	1.07	9.48	2.64	9.71	3.08	9.87	2.72
7	13.77	-2.89	8.86	1.26	10.52	2.89	10.76	3.03	10.88	2.68
8	13.25	-2.97	10.25	1.42	11.62	2.99	11.86	2.92	11.94	2.80
9	15.50	-3.13	11.64	1.62	12.77	2.95	13.02	3.02	13.05	2.81
10	15.10	-3.08	13.04	1.78	13.99	2.94	14.23	3.11	14.23	2.84
11	22.78	-3.02	14.46	1.92	15.27	3.11	15.50	3.11	15.46	3.06
12	23.40	-3.59	15.88	2.11	16.61	3.23	16.83	3.25	16.75	3.15
13	24.45	-3.36	17.33	2.28	18.01	3.30	18.20	3.31	18.10	3.05
14	29.95	-3.98	18.78	2.50	19.45	3.45	19.63	3.33	19.49	3.04
24	35.67	-5.42	34.40	4.24	36.16	5.51	36.10	4.03	35.68	4.20
average	20.11	2.65	12.22	1.40	14.18	2.63	14.26	2.53	14.30	2.45

Supplementary Table 15: CV (in %) and MRE (in %) calculated for time-dependent parameters at different time points (t). The simulation was done with $n = 100$, $i = 3000$, $j = 15$ (E15, Table 14), and max Robust loss. Since the model forces $c_0^{\text{caf}} = 0$ no CV and MRE were calculated for this instance.

t	V_{sweat}		c^{caf}		c^{par}		c^{bro}		c^{phy}	
	CV	MRE	CV	MRE	CV	MRE	CV	MRE	CV	MRE
0	19.93	-4.24	-	-	19.15	4.18	18.84	3.69	18.93	3.40
0.25	24.87	-5.63	21.70	5.31	18.93	4.32	18.56	3.78	18.65	3.49
0.5	18.23	-5.39	16.46	4.22	17.68	4.48	17.31	3.84	17.41	3.86
0.75	19.09	-4.21	12.51	3.44	15.89	4.44	15.55	3.98	15.66	3.98
1	22.55	-3.58	9.51	2.66	14.01	4.33	13.71	4.04	13.82	4.03
1.5	15.59	-2.81	5.55	1.67	10.84	3.77	10.64	3.63	10.72	3.93
2	12.67	-2.33	3.50	0.74	8.89	3.11	8.78	2.96	8.80	3.33
3	15.37	-1.74	3.25	0.63	7.94	2.42	7.94	2.30	7.85	2.60
4	13.77	-2.02	4.60	1.25	8.70	2.45	8.77	2.35	8.62	2.91
6	14.66	-3.38	7.41	2.29	10.80	2.71	10.98	2.88	10.81	3.37
8	16.49	-4.03	10.19	3.24	12.79	3.28	13.06	3.36	12.92	3.70
24	32.77	-9.38	35.33	10.75	35.51	10.43	35.34	10.37	35.50	10.05
25	38.29	-10.13	37.14	11.23	37.37	10.90	37.16	10.81	37.33	10.46
26	36.59	-10.95	38.98	11.67	39.29	11.34	39.03	11.17	39.22	10.95
27	36.06	-11.22	40.86	12.11	41.26	11.87	40.95	11.58	41.16	11.32
average	22.46	5.40	17.64	5.09	19.94	5.60	19.77	5.38	19.83	5.43

Supplementary Table 16: CV (in %) and MRE (in %) calculated for simulations with different assumed experimental errors (σ_ϵ). Simulations were done with $n = 100$, $i = 300$, $j = 20$, and Max Robust loss.

ϵ	1%		10%		20%		30%	
	CV	MRE	CV	MRE	CV	MRE	CV	MRE
k_1	9.13	0.16	25.56	1.48	39.74	2.5	54.41	10.01
k_2	4.18	0.07	19.08	4.07	28.05	6.69	35.02	7.25
k_3	4.3	0.63	19.08	4.19	28.33	4.46	38.66	13.54
k_4	4.09	0.63	18.44	2.24	30.0	8.41	41.49	15.68
k_5	41.72	-21.92	41.22	-13.41	44.02	-5.88	51.09	-9.48
k_6	13.04	-6.48	22.63	0.92	33.22	6.48	38.01	4.88
k_7	15.87	-8.15	28.69	3.32	38.8	5.01	54.46	20.29
k_8	16.1	-7.61	26.86	2.84	43.44	7.97	53.21	20.48
c_0^{par}	5.17	-0.07	17.64	-0.73	24.69	2.81	36.16	3.66
c_0^{bro}	5.39	-0.5	18.17	0.48	24.8	3.44	33.61	1.34
c_0^{phy}	5.24	-0.58	18.11	-0.13	24.29	0.84	33.56	-0.13
V_{sweat}	18.12	-2.76	19.66	-2.57	24.05	-4.64	30.15	-5.6
c^{caf}	12.64	3.09	12.88	1.51	13.51	0.52	15.2	-0.52
c^{par}	12.61	2.88	14.49	2.91	18.07	3.37	21.98	3.26
c^{bro}	12.63	2.88	14.48	3.2	17.63	2.77	21.65	3.51
c^{phy}	12.66	2.86	14.61	2.89	17.73	3.07	21.84	3.93
average	12.06	3.83	20.72	2.93	28.15	4.3	36.28	7.72

Supplementary Table 17: Comparison of CV (in %) and MRE (in %) calculated with and without V_{sweat} normalization. Simulations were done with $n = 100$, $i = 3000$, $j = 20$, and max Robust loss. We point out that the simulation without V_{sweat} normalization produced substantially more outliers (relative errors above 1000% for the V_{sweat}). For all other simulations the fraction of outliers was below 0.1% (e.g. here 0.01% with V_{sweat} normalization), without V_{sweat} normalization it increased to 0.73%.

	without V_{sweat} normalization		with V_{sweat} normalization	
	CV	MRE	CV	MRE
k_1	62.75	6.21	25.21	1.69
k_2	32.2	1.54	17.56	3.01
k_3	35.66	4.03	17.65	3.81
k_4	35.77	2.83	17.85	3.73
k_5	71.75	1.38	38.12	-8.55
k_6	41.37	-0.91	22.35	1.09
k_7	53.14	2.95	27.11	3.18
k_8	52.87	1.45	26.8	2.94
c_0^{par}	21.37	1.68	17.12	1.07
c_0^{bro}	22.56	2.50	16.95	0.45
c_0^{phy}	22.8	3.13	17.02	0.56
V_{sweat}	99.76	-0.96	20.95	-2.45
c^{caf}	19.50	-0.90	12.22	0.87
c^{par}	17.22	0.50	14.03	2.51
c^{bro}	17.01	1.34	14.12	2.51
c^{phy}	17.02	1.33	14.15	2.40
average	38.92	2.10	19.95	2.55

2 Supplementary Figures

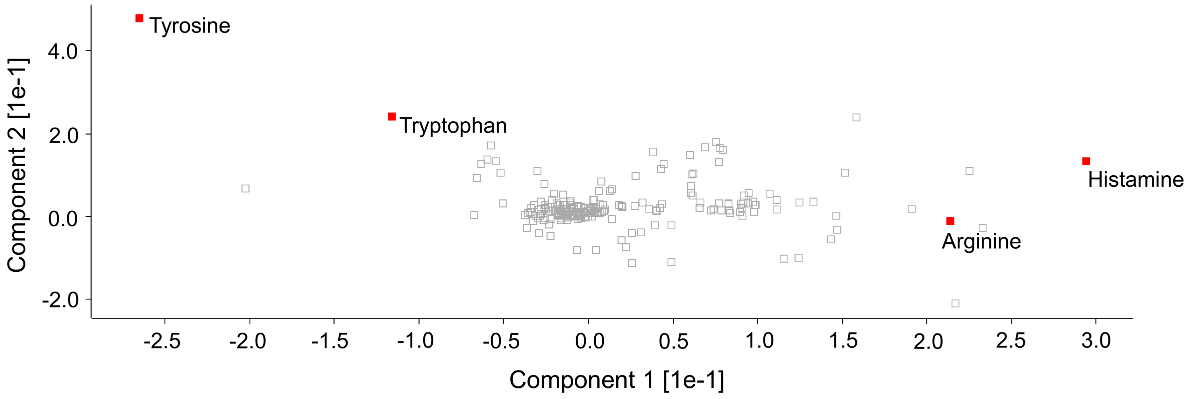

Supplementary Figure 1: Loading plot for the PCA depicted in Figure 1d, showing the strong influence of histamine, arginine, tyrosine and tryptophan on components 1 and 2.

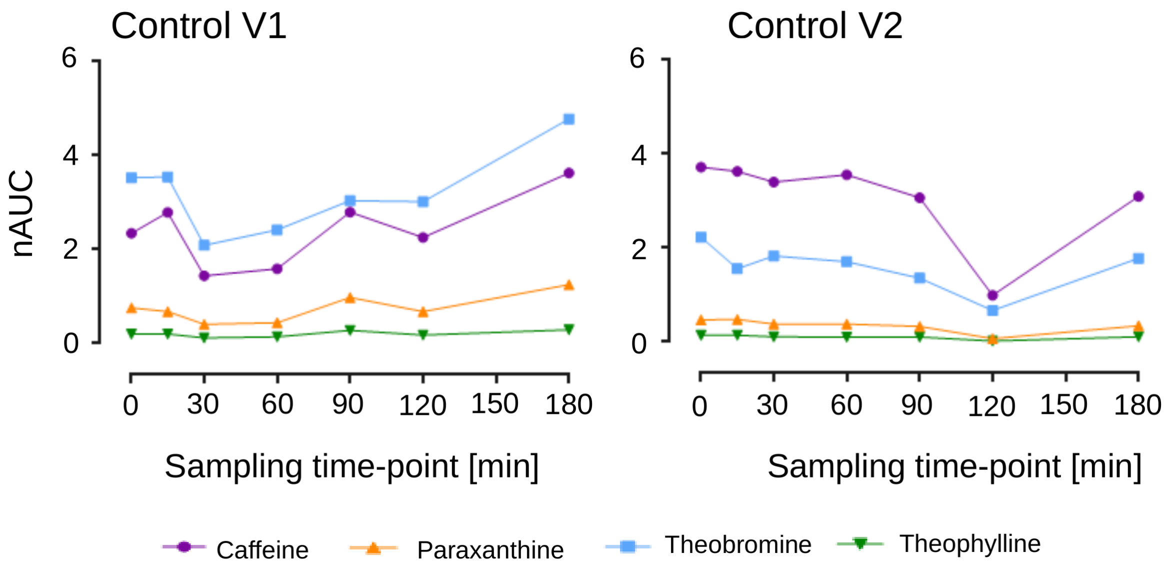

Supplementary Figure 2: Metabolite profiles of donors having fasted caffeinated foods and drinks for at least 12 h. There is no significant increase in xenobiotic metabolite levels. Small fluctuations are based on different sweat amounts. nAUC, normalised area under the curve.

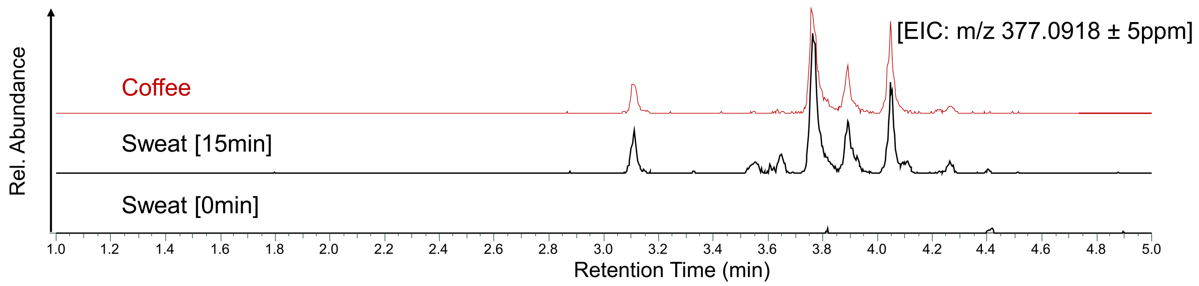

Supplementary Figure 3: Similarity of extracted ion chromatograms (EIC) of the unidentified feature 377.0918 and its isomers regarding the source (coffee) and the detection in finger sweat samples 15 minutes (15min) after coffee consumption. The corresponding finger sweat sample collected just before coffee consumption (0min) served as negative control.

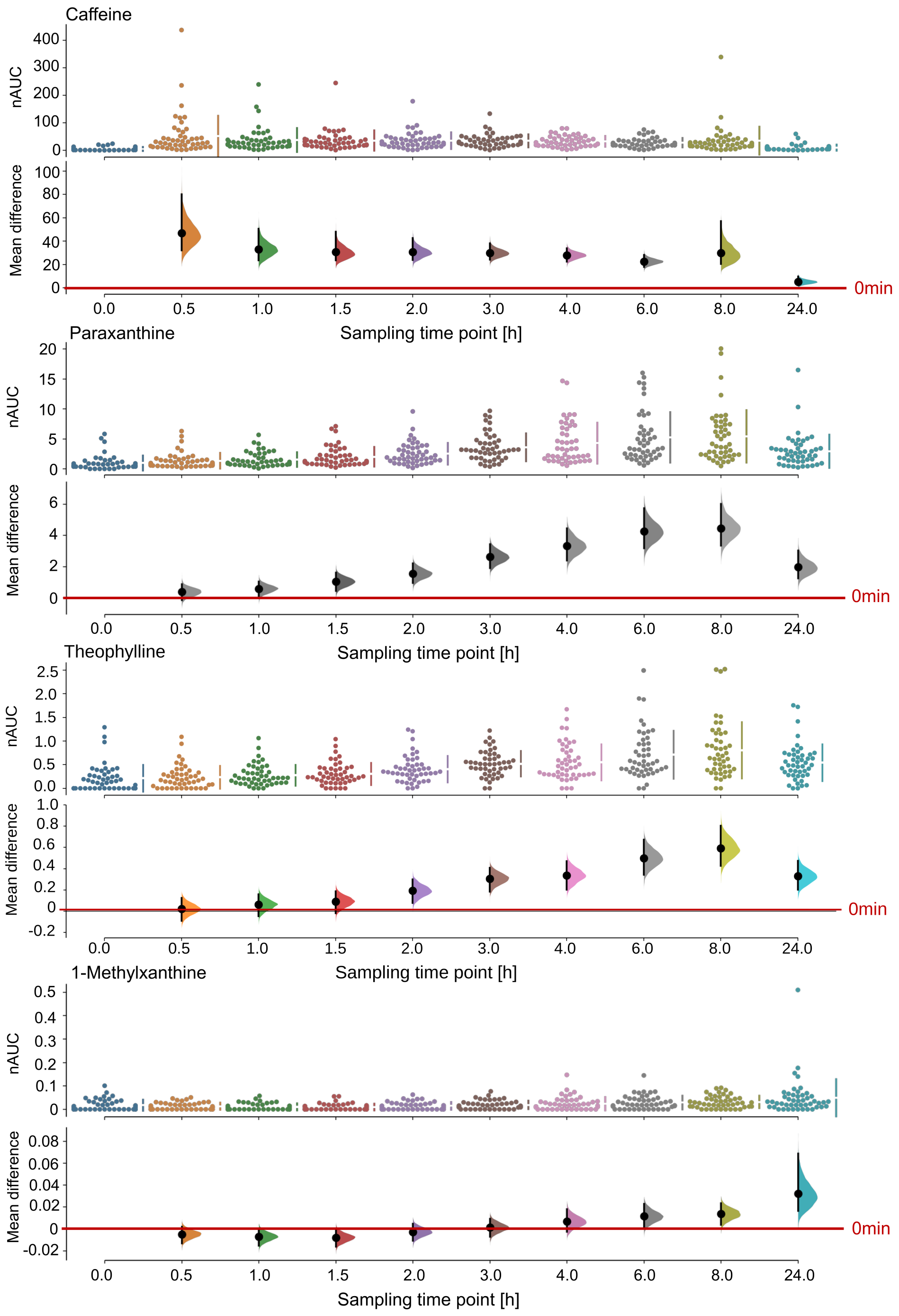

Supplementary Figure 4: Effect size plots with data from 47 individuals (n=47 for 10 time-points) for caffeine, paraxanthine, theophylline and 1-methylxanthine. A shared-control estimation plot was performed, which presents the mean differences between a single control (time-point before consumption, red line) and each of the intervention groups. All nAUC datapoints are presented as a swarmplot (top). The effect size is presented as a bootstrap 95% confidence interval (bottom), where the effect size is displayed to the right of the raw data, and the mean of the test group is aligned with the effect size. Exact values for the mean difference, the lower and upper limits as well as the p-values can be found in the Source Data sheet. nAUC, normalised area under curve.

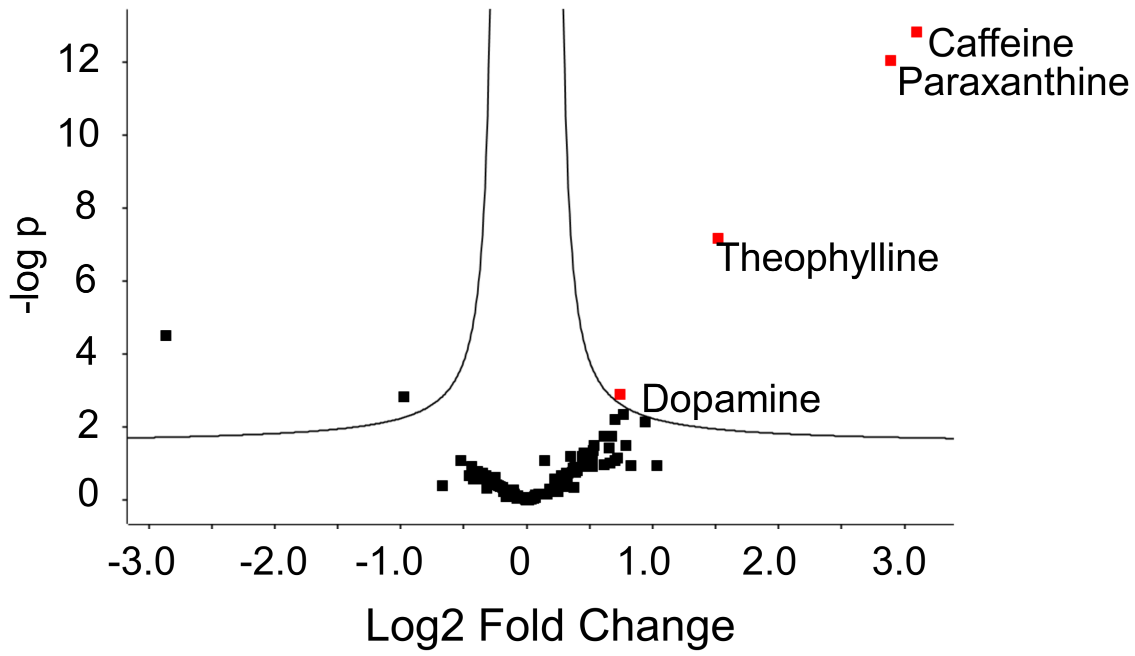

Supplementary Figure 5: Metabolic changes in 27 donors 5 h post ingestion of a 200 mg caffeine capsule demonstrated by a two-sided volcano plot. The -log p value for caffeine is 12.82, for paraxanthine 12.05, for theophylline 7.16 and for dopamine 2.91. The false discovery rate was set to 0.05 and the minimal fold change (s0) to 0.1.

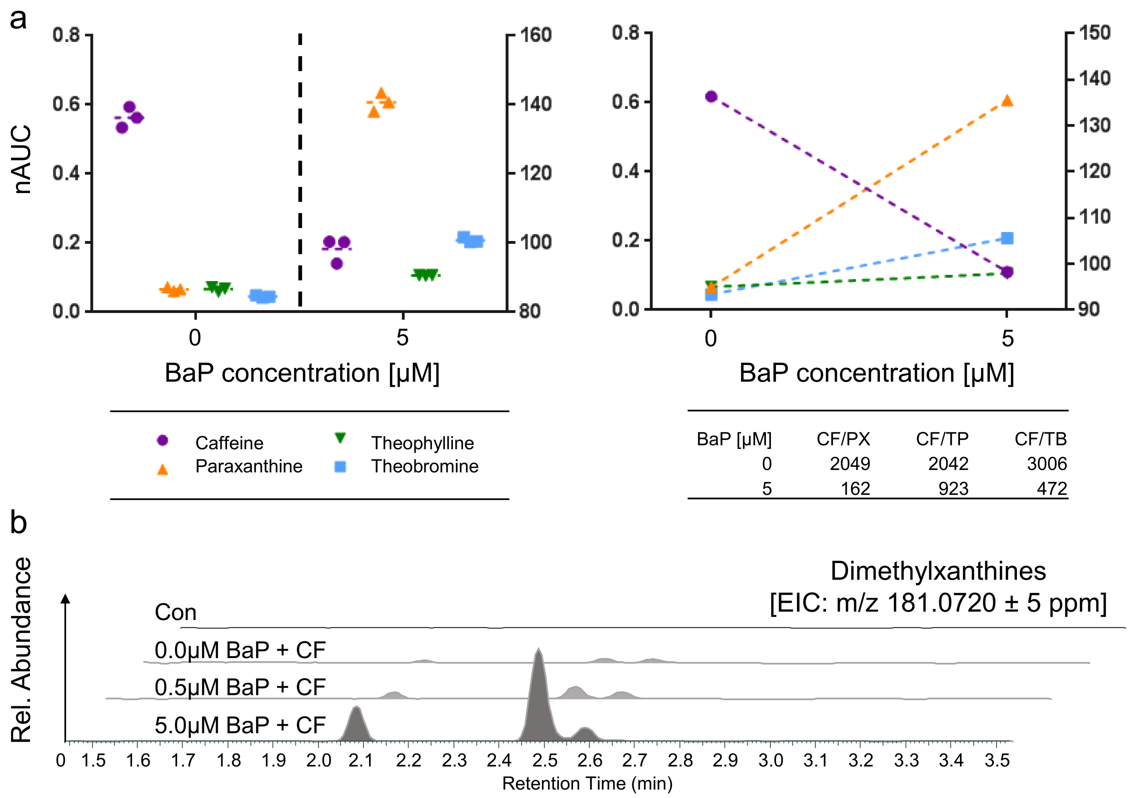

BaP [µM]	CF/PX	CF/TP	CF/TB
0	2049	2042	3006
5	162	923	472

Supplementary Figure 6: Cell culture experiment with HepG2 cells. **a** HepG2 cells treated with 100 µM caffeine and either no or 5 µM benzo-[a]-pyrene, a known inducer of cytochrome P450 enzymes (e.g. CYP1A1). The concentrations of caffeine and its primary metabolites obtained from cell supernatants is depicted using three independent replicates (left). The decrease of the caffeine concentration and an increase in metabolite concentrations when treated with benz-[a]-pyrene is highlighted (right), demonstrating that the treatment with benzo-[a]-pyrene led to a more efficient transformation of caffeine to its metabolites via the induction of cytochrome P450 enzymes. Pre-treatment with benzo-[a]-pyrene caused an enhanced production of paraxanthine, indicating that cellular metabolic pathways may be modulated chemically. Different metabolite ratios are shown below. **b** Metabolite levels increase in supernatants of HepG2 cells with increasing benzo-[a]-pyrene concentration. Primary caffeine metabolites were absent in control cells without caffeine treatment both in the cells with and without pre-treatment with benzo-[a]-pyrene (5 µM). BaP = benzo-[a]-pyrene; CF = caffeine; nAUC = normalised area under the curve; PX = paraxanthine; TB = theobromine; TP = theophylline. EIC = extracted ion chromatogram; Rel. = relative.

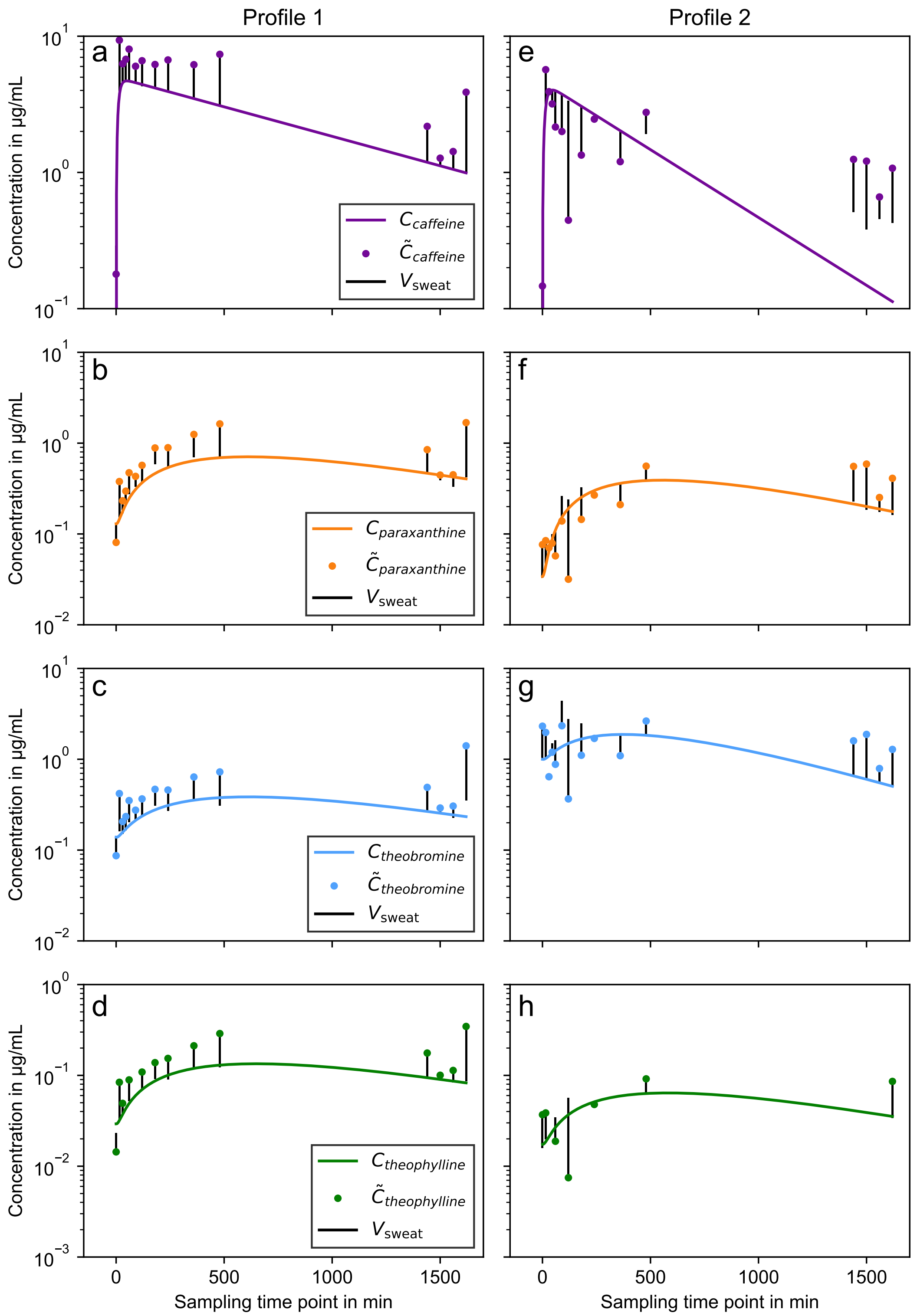

Supplementary Figure 7: Visual representation of the influence of $V_{\mathbf{sweat}}(t)$. Concentration time-series of caffeine, paraxanthine, theobromine and theophylline (first to last row in that order) for profile 1 and profile 2 (left and right column respectively). The symbols refer to the measured values, the coloured lines refer to the fitted values and the black bars indicate how much of the difference is described by $V_{\text{sweat}}(t)$. Note that the y-axis is logarithmic.

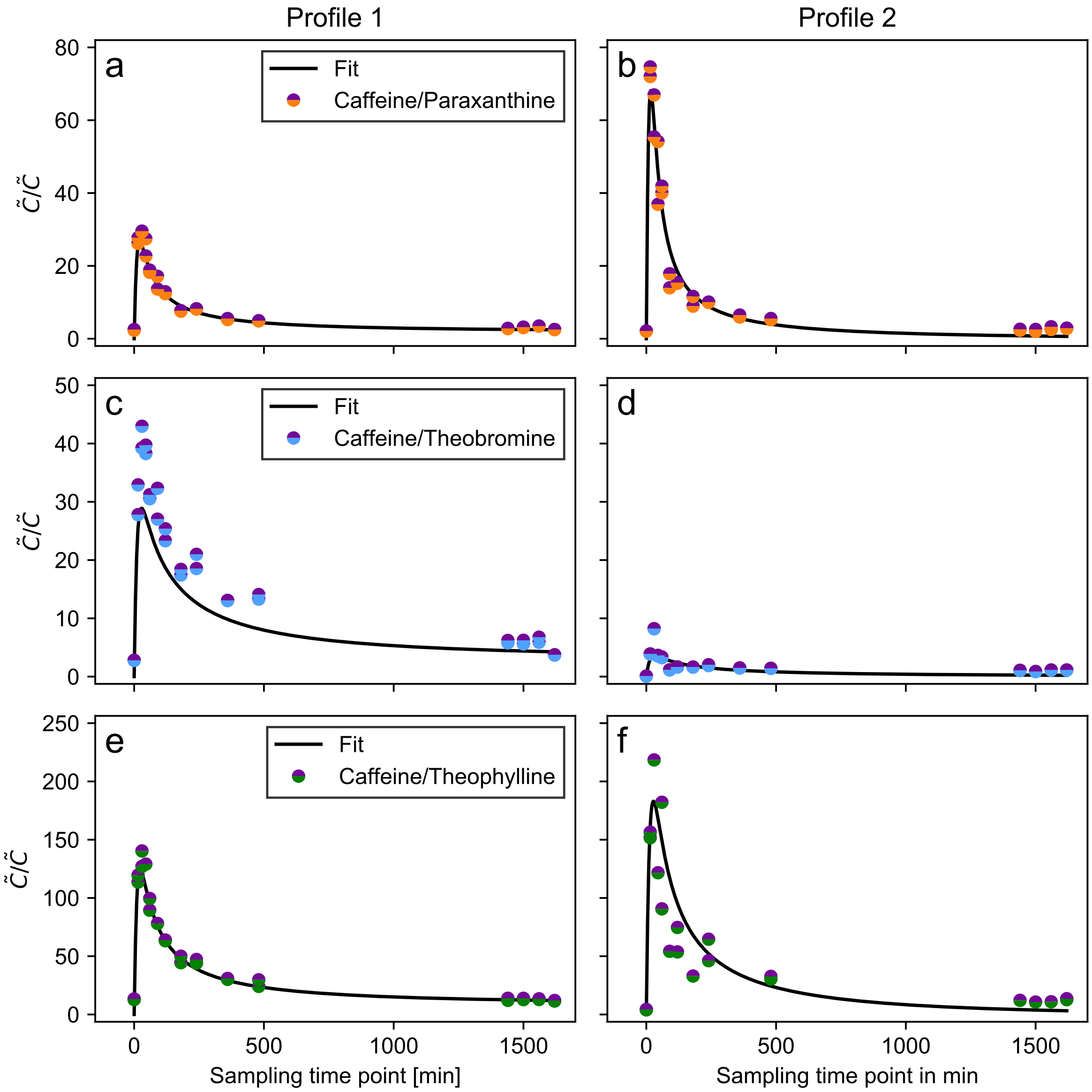

Supplementary Figure 8: The sweat volume cancels out upon division of two metabolites. The noise introduced by the different sweat volumes cancels out upon division of the concentration time series of two metabolites as shown here for caffeine and its major degradation products for profile 1 and 2 (left and right column respectively). The fit curve corresponds to the division of the fitted time-curves of the respective metabolite pair.

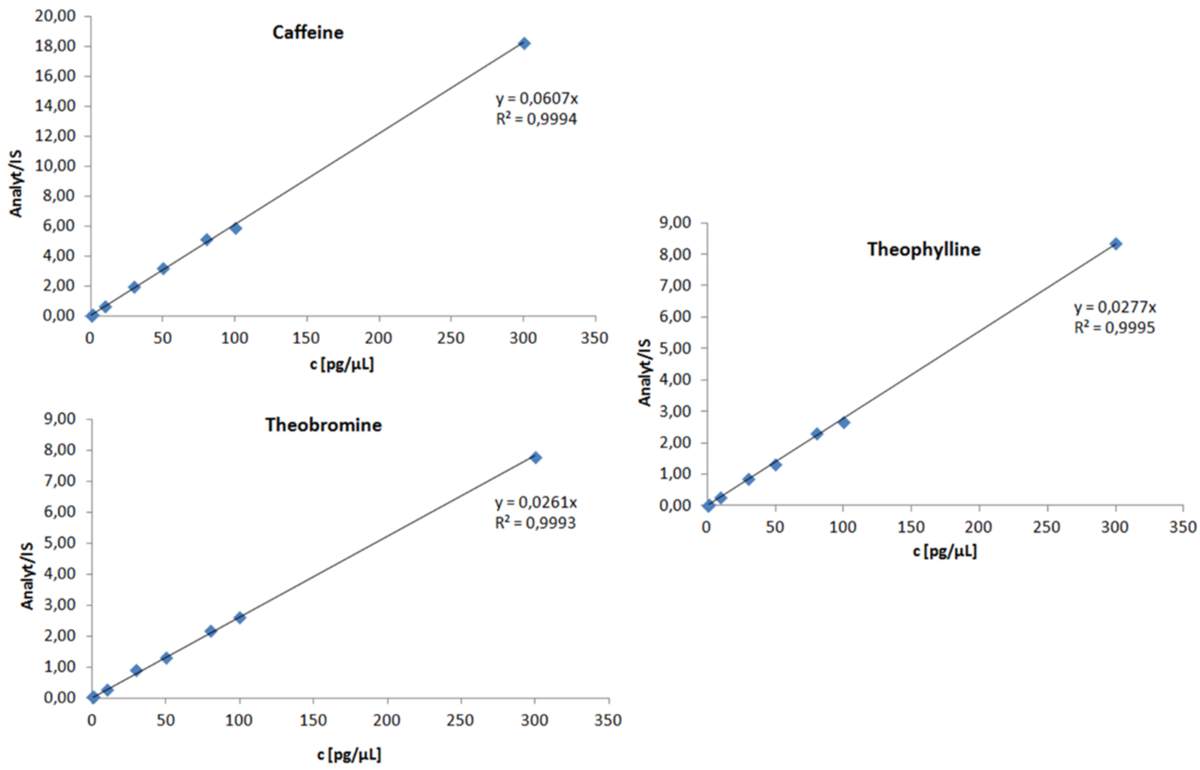

Supplementary Figure 9: Calibration curves for caffeine, theobromine and theophylline in eight concentration levels in the range of 0.5–300 pg/µL (0.25–150 pg/µL on column) obtained using the microfluidics-based chip-cube separation system coupled to a triple quadrupole mass spectrometer with their corresponding correlation factors (R^2) are shown. IS = internal standard.

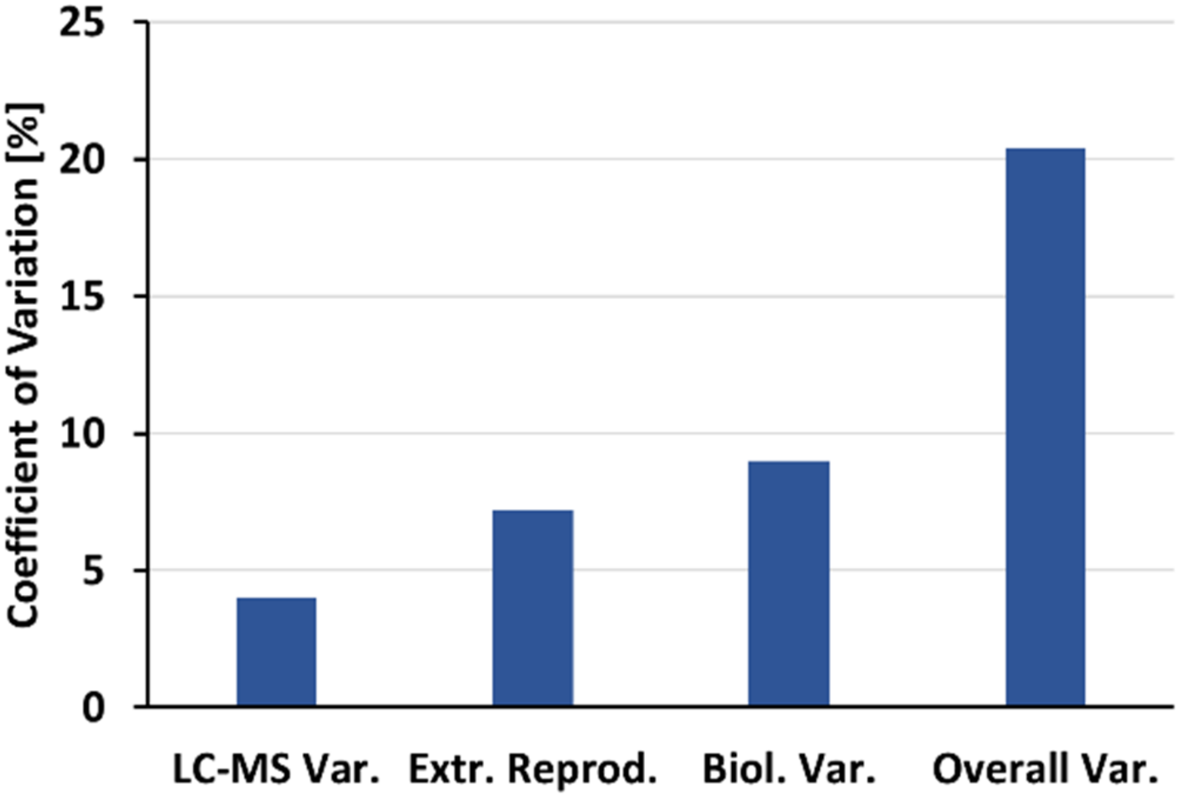

Supplementary Figure 10: Coefficients of variation of caffeine quantification from the fingertips using the microfluidic-based chip-cube separation system coupled to a triple quadrupole mass spectrometer. The LC-MS variation represents the coefficients of variations of 3 technical replicates. The extraction reproducibility represents the coefficient of variation of 3 biological extractions, each with 3 technical replicates. The biological variation indicates the coefficients of variation of the average caffeine amount after 5 h of coffee intake of all the donors. The overall variation represents the coefficients of variations of all technical and biological replicates of 5 donors after 5 h of coffee intake.

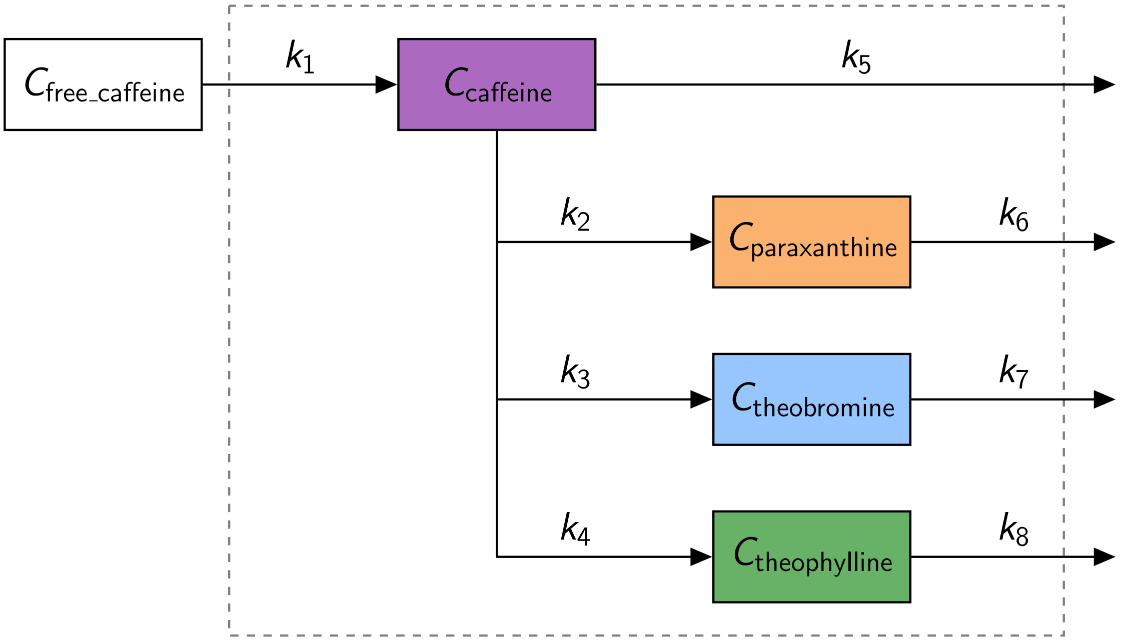

Supplementary Figure 11: Metabolic network of caffeine absorption and degradation. The system boundary (dashed line) represent the human body.

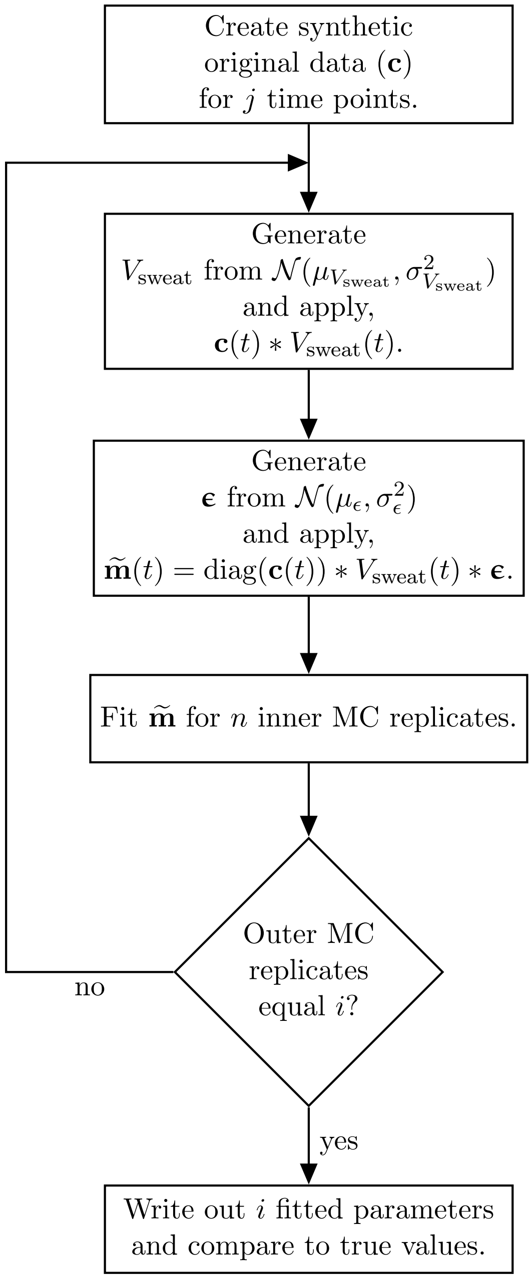

Supplementary Figure 12: Simulation work flow. Work flow scheme of error estimation simulations with the sweat volume $V_{\text{sweat}}(t)$ and experimental error ϵ as confounding factors of the true values.

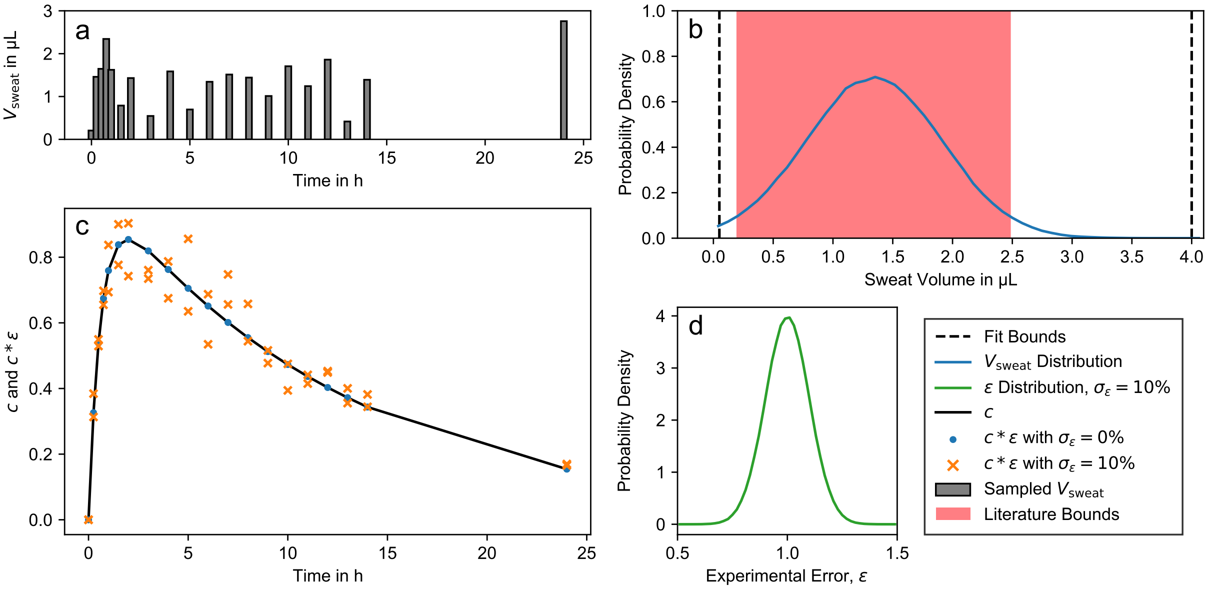

Supplementary Figure 13: Simulation data creation. **a** Example of the sampled V_{sweat} values for the time points τ. **b** Probability density of the V_{sweat} values of all simulated runs. The distribution follows a Gaussian $\mathcal{N}(1.34\,\mu\text{L}, 0.57^2\,\mu\text{L})$ truncated at 0. The range reported in literature is shown as red area, the bounds of V_{sweat} parameters in the fitting procedure are given as black dashed lines. **c** Representation of the data processing work flow. Firstly, we assume true data (black line), then we apply an experimental error, ϵ (0%: blue dots, 10%: orange crosses). The application of V_{sweat} is not shown. **d** Probability density of the (relative) experimental error of 10% following a Gaussian $\mathcal{N}(1, 0.1^2)$.

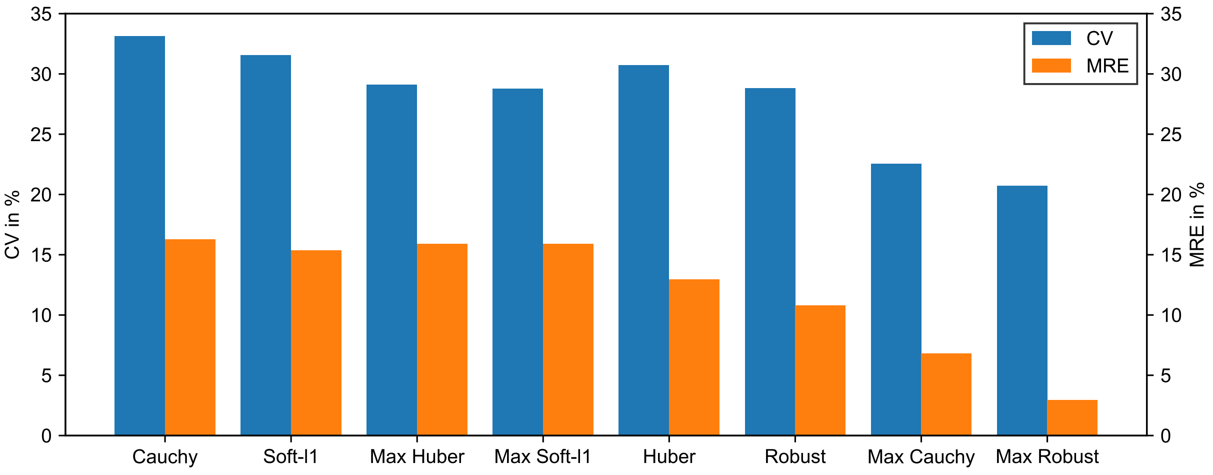

Supplementary Figure 14: Influence of different loss functions on the fitting error. Averages of CV and MRE over all model parameters and concentrations are plotted. 'Max' in front of the loss name indicates that the maximum of either absolute or relative error was used for every time point. The losses are sorted according to their overall performance (= CV + MRE). The raw data is given in Supplementary Table 10.

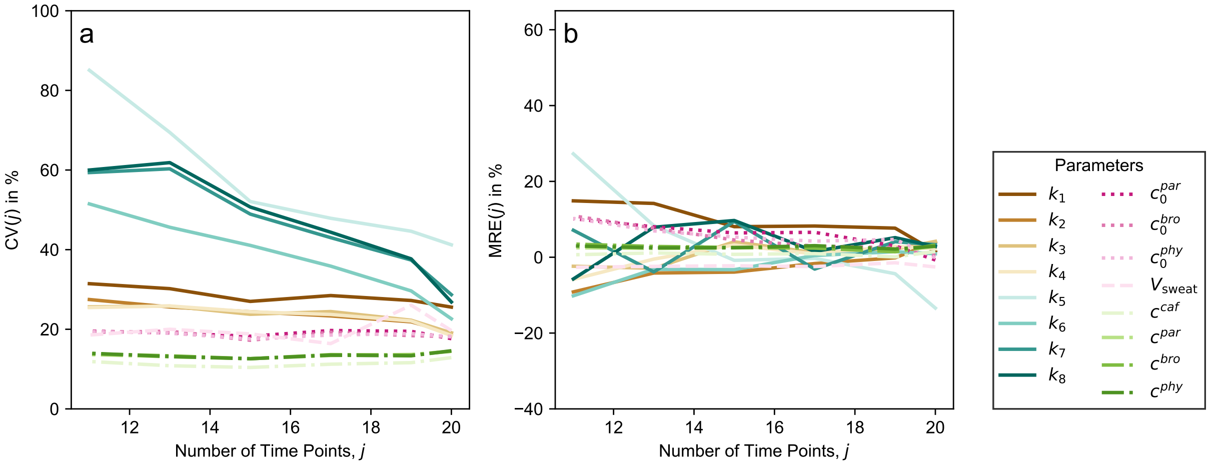

Supplementary Figure 16: Influence of the number of measured time points (j) on the fitting error. **a** Change of coefficient of variation (CV) and **b** median of relative error (MRE) of simulations with j time points. Note the difference in scale of the y-axis. The raw data is given in Supplementary Table 13. Simulation E15 is not shown.

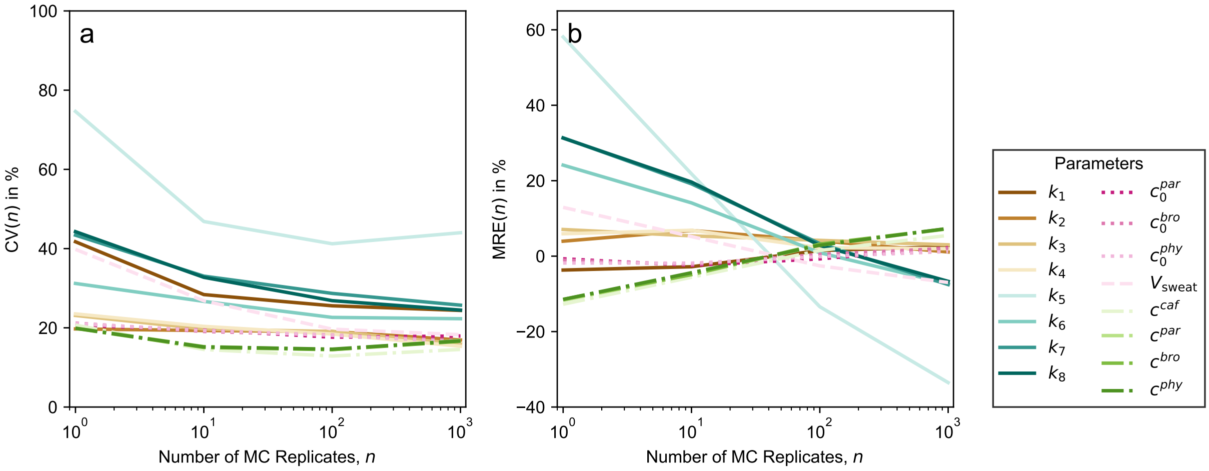

Supplementary Figure 15: Influence of the number of MC replicates (n) on the fitting error. **a** Change of coefficient of variation (CV) and **b** median of relative error (MRE) of simulations with n MC replicates. Note the difference in scale of the y-axis. The raw data is given in Supplementary Table 11.

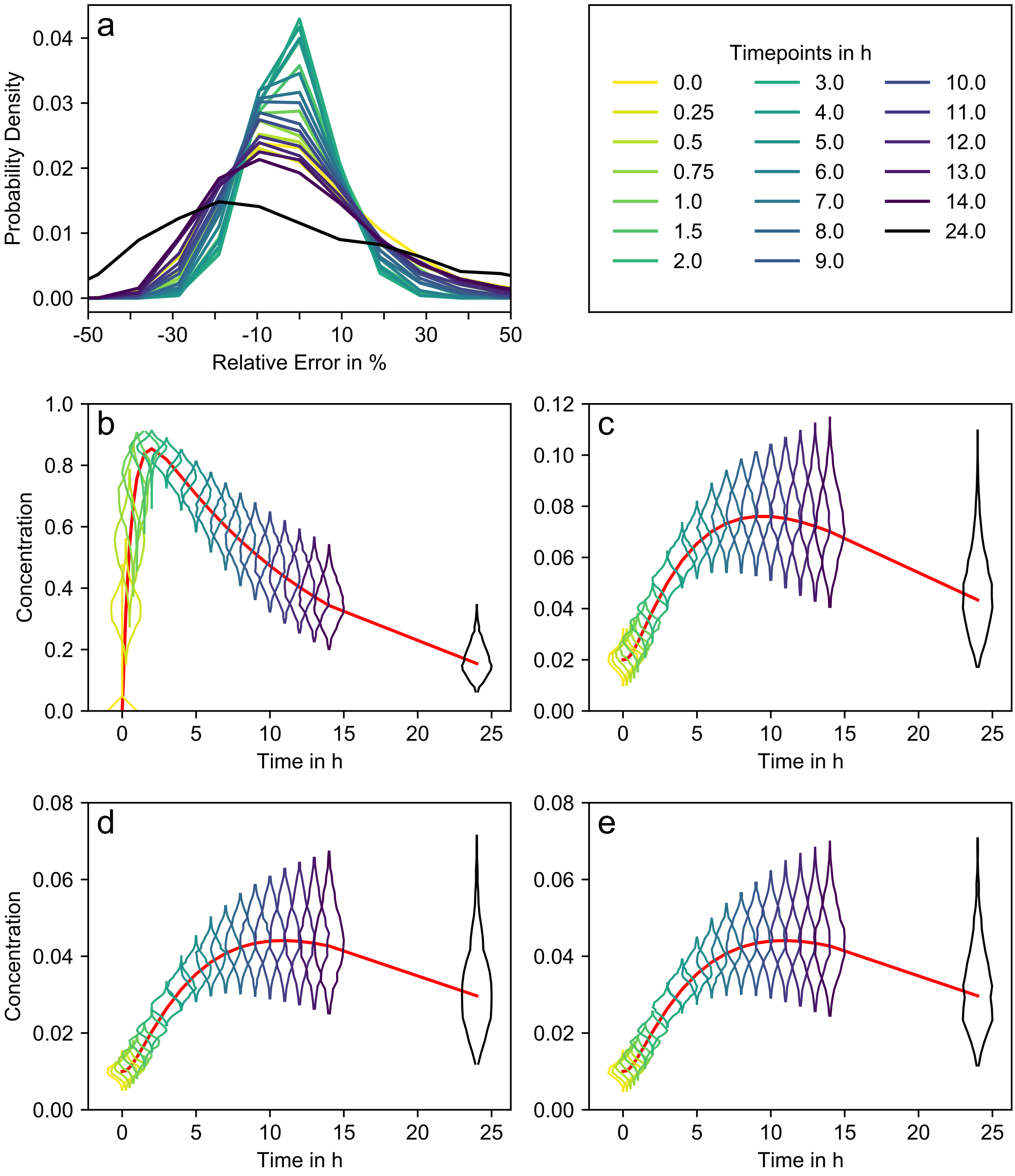

Supplementary Figure 17: Time-dependent error distributions. **a** The error distribution of V_{sweat} clearly widens for later time points. The calculated CVs and MREs are shown in Supplementary Table 14. Error distribution of unit-less concentration of caffeine (**b**), paraxantine (**c**), theobromine (**d**), and theophylline (**e**) at different time points shown as violin plots. No curve smoothing was performed. The true values (**c**) are shown as red line. The calculated CVs and MREs are given in Supplementary Table 14. Note the different scales of the y-axis.

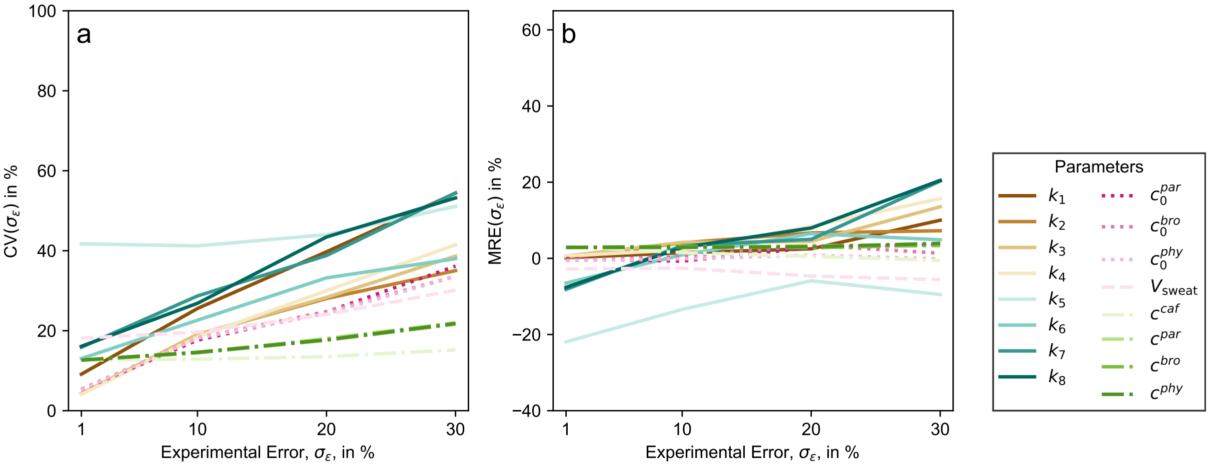

Supplementary Figure 18: Influence of experimental error (σ_ϵ) on the fitting error. **a** Change of coefficient of variation (CV) and **b** median of relative error (MRE) of simulations depending on the assumed experimental error σ_ϵ. The raw data is given in Supplementary Table 16.

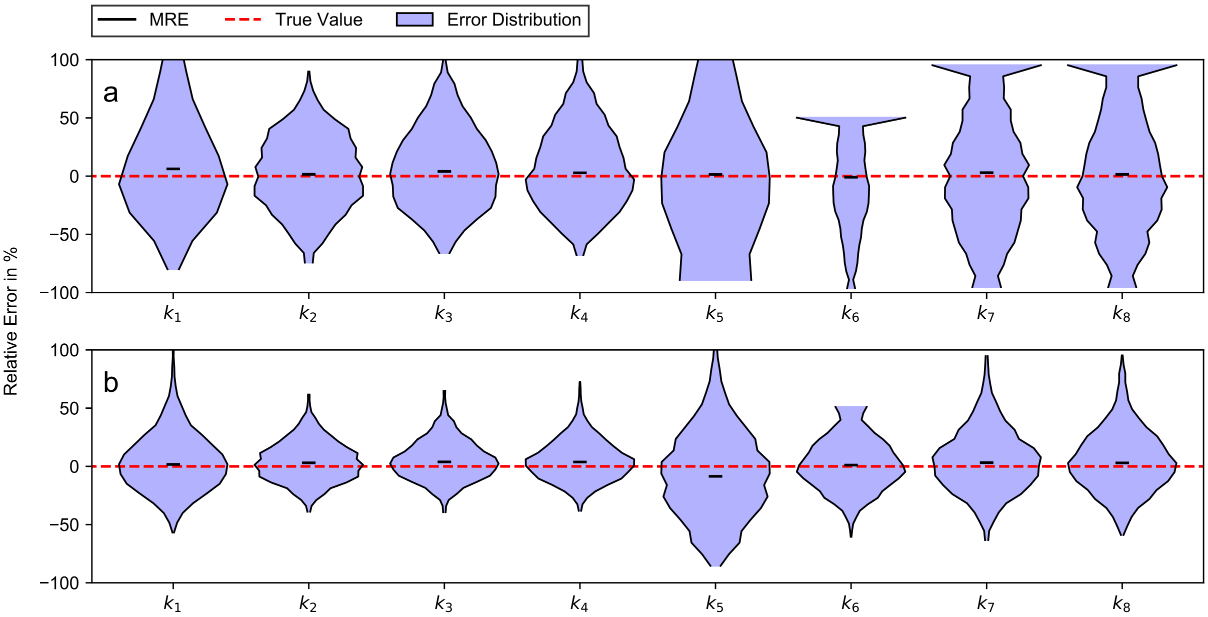

Supplementary Figure 19: Influence of V_{sweat} normalisation on the fitting error. Fitting error distribution without (**a**) and with (**b**) accounting for a variable sweat volume V_{sweat}. The raw data is given in Supplementary Table 17. The distortion of the distributions for k_6 to k_8 in panel **a** are due to the fitting bounds limiting greater values.

3 Supplementary Note 1: Caffeine metabolisation in HepG2 cells

3.1 Cell culture conditions

HepG2 cells (purchased from ATCC, HB–8065) were cultivated in Eagle's minimum essential medium (EMEM, Gibco) with 10% fetal calf serum (FCS, Gibco), 1% penicillin/streptomycin (Sigma-Aldrich, Austria), 2 mM L-glutamine (Sigma-Aldrich, Austria) and 1% MEM non-essential amino acids (Gibco). Cultivation was performed in a humidified atmosphere at 37 °C and 5% CO_2. For assessing caffeine metabolisation, HepG2 cells were seeded in 6-well plates with cell growth surface for adherent cells (Sarstedt, Austria) at a density of 7×10^5 cells/well in 1.5 mL complete medium. Cells were left over night. The growth medium was exchanged and the samples incubated for 24 h with either solvent control (con) or 100 µM caffeine. A caffeine stock solution (20 mM) was prepared in PBS. Additionally, HepG2 cells were pre-treated with benzo-[a]-pyrene (BaP, Sigma) at 0.5 µM or 5 µM for 6 h to induce cytochrome P450 enzymes.[4] A BaP stock solution (20 mM) was prepared in DMSO. After exchanging the medium, cells were again treated with solvent control (con) or caffeine (100 µM) and incubated for 24 h. All experiments were performed in triplicates.

3.2 Metabolite Extraction

Supernatants were precipitated in cold methanol (100%, VWR) 1:5 *v/v*. The methanolic solution contained dopamine-D4, melatonin-D4 (both Santa Cruz Biotechnology) and N-acetyl-serotonin-D3 (Toronto Research Chemicals BIOZOL) as internal standards at concentrations of 120 pg µL^{-1}. Adherent cells were washed once with cold PBS (1 mL) and wash solutions were immediately removed under suction with a Pasteur pipette. Then, the extraction solution (500 µL) was added onto the cells. The extraction solution consisted of 80% cold methanol (VWR), including the internal standards dopamine-D4, melatonin-D4 (both Santa Cruz Biotechnology) and N-acetyl-serotonin-D3 (Toronto Research Chemicals BIOZOL) at concentrations of 100 pg µL^{-1}. Each well was processed at a time. The 6-well plate was snap-frozen in liquid nitrogen. After thawing, cells were scraped and the resulting suspension transferred into Eppendorf tubes and stored at −20 °C until the time of analysis. Prior to the analysis, the samples were thawed and centrifuged (15000 g, 10 min). The supernatant (400 µL) was transferred into HPLC glass vials (Macherey-Nagel GmbH Co.KG, for LC-MS/MS analysis) and dried under a gentle stream of nitrogen. Dried samples were then dissolved in the initial LC eluent composition (120 µL), transferred into glass vials equipped with a V-shaped glass insert and assessed with the same untargeted approach as finger sweat samples (s. Methods).

4 Supplementary Note 2: Targeted approach using multiple reaction monitoring (MRM)

4.1 Chemicals

Caffeine and formic acid were obtained from Fluka. Caffeine-D9, paraxanthine, theobromine and theophylline were obtained from Sigma-Aldrich. Ultrapure water was obtained from a Millipore system (18.2 MΩ, 185 UV, Millipore), all other solvents were purchased from VWR (LC-MS grade). All chemicals and solvents were used as received.

4.2 Standard Solutions and Calibration Samples

The stable isotope-labelled caffeine-D9 was used as the internal standard. It was spiked to all samples in a concentration of 10 pg μL^{-1}. Additionally, a standard mixture containing caffeine, theobromine and theophylline (each 100 pg μL^{-1}) was measured every thirtieth sample as a quality control. The limit of detection (LOD) was defined as the lowest concentration of the analyte that can be detected with a signal-to-noise ratio of 3:1. The lower limit of quantification (LLOQ) was defined as the lowest concentration of the analyte that can be detected with a signal-to-noise ratio of 10:1.

4.3 Samples

The temporal evolution of caffeine, theobromine, theophylline and paraxanthine in fingertips of five volunteers was investigated. This was the suggested number of volunteers by power analysis in order to obtain statistical relevant data (calculated using R-studio with a 10% error rate and a significance criterion of 0.05). Two female and three male volunteers were recruited between 25 and 30 of age. All volunteers were non– to moderate coffee consumers. The volunteers were asked to fast caffeine-containing products for 12 h before the start of each experiment. Subjects presented on 8 am on the study day before the first cup of coffee (equivalent to a double espresso, approx. 80 mg caffeine). Samples from the fingertips were collected before and 1, 3 and 5 h after coffee consumption. The experiment was performed independently on three different days.

4.4 Sample Preparation

Sample collection was performed similarly as described in the methods section of the main text. The filter paper was transferred into an Eppendorf tube and metabolites were extracted with acetonitrile (500 µL). First, the extraction solution was vortexed for 1 min and then stirred in am Eppendorf Thermomixer comfort 1.5 mL (40 °C, 1400 rpm, 10 min). This process was repeated twice and finally the mixture was centrifuged (10 min, 20000 rpm). A fraction of the sample solution (250 µL) was transferred into a fresh Eppendorf tube. The samples were dried under a flow of nitrogen. The dried residues were reconstituted in water (250 µL) containing 0.2% formic acid. The extracted samples were sonicated (10 min) and transferred into 96-well plates for analysis.

4.5 Targeted LC-MS/MS

Targeted LC-MS/MS experiments were performed using a nano- and cap-pump (1260 Infinity, Agilent) together with a microfluidics-based separation system (Chip-Cube, Agilent) hyphenated to a triple quadrupole mass spectrometer (Agilent 6490). Liquid Chromatography. The analyte separation was performed using a chip-based integrated sample enrichment, separation and nanoESI sprayer tip (UHC-CHIP II, ZORBAX 80SB-C18, 5 µm, 25 mm×75 µm enrichment column and 150 mm×75 µm separation column, Agilent). The injection volume was 0.5 µL. The autosampler was held at 4 °C. Mobile phase A was aqueous solution (0.2% FA) and mobile phase

B was acetonitrile (0.2% FA). The gradient included a total run time of 25 min using a flow rate of 400 nL min^{-1}. A stepped-gradient was applied by starting with 0% B and increasing to 8% B within 0.1 min. The mobile phase B was linearly increased to 20% (3 min) and then to 80% (5 min), which was held constant for 4 min. Then, the mobile phase B was decreased to 0% and the system allowed to equilibrate for further 16 min. A mixture of isopropanol, acetonitrile, methanol and water (1:1:1:1) was used for needle washes.

4.6 Mass Spectrometry

The analytes were detected via multiple reaction monitoring (MRM) of three different transitions per molecule with a cycle time of 0.8 s and a dwell time of 50 ms per transition (Supplementary Table 4). Typical MS parameters were as follows: capillary voltage -1.7 to −1.9 kV, gas flow 13 L min^{-1}, dry gas temperature 200 °C. Experiments were performed and evaluated using Mass Hunter B.06.00 (Agilent).

5 Supplementary Note 3: Mathematical model

In this section we present the mathematical model used to described the metabolic network of Supplementary Figure 11 (Figure 5a in main manuscript). A list of symbols used in this section is given in Supplementary Table 6. Briefly, the model describes concentration time series of one the ingested free caffeine ($C^{\text{free caf}}$) and four sweat metabolites (C^{caf}, C^{par}, C^{bro}, C^{phy}) within the constraints of the following assumptions:

- caffeine metabolism can be described by mass-action kinetics in a one-compartment body model,
- the uptake of the ingested caffeine ($C^{\text{free\_caf}}$) is instantaneous (i. e. no lag time between ingestion and absorption into the body),
- the steady-state volume of distribution (V_{D}) of all four internal metabolites is instantaneously reached and time-independent,
- concentration enrichment due to an increase in the water fraction from blood to sweat and dilution through the inability of bound caffeine to diffuse as described by ref. [5] are approximately equal and cancel each other out,
- the apparent metabolite concentrations are proportional to the sweat volume, and finally,
- sweat volumes are time-dependent and constant across all metabolites.

In the following equations, we differentiate between three types of variables: (i) true variables, denoted by capital letters (e.g. C, which denotes a measure, non-obscured concentration), (ii) apparent variables, denoted by a tilde (e.g. $\tilde{C}$), wich denotes a measured concentrations that is obscured by an unknown sweat volume, V_{sweat}, and (iii) true specific variables, denoted as lower-case letters, such as normalised concentrations c that have been divided by another variable. Additionally, we use vector notation to collectively refer to a set of metabolites, e.g. $\mathbf{C} = \begin{pmatrix} C^{\text{caf}} & C^{\text{par}} & C^{\text{bro}} & C^{\text{phy}} \end{pmatrix}^{\text{T}}$, which denotes the vector of (true) concentrations for caffeine, paraxanthine, theobromine, and theophylline, respectively.

The fluxes described by the metabolic network can be separated into three, fluxes into the system ($\mathbf{J_{in}}$), rearrangements of internal fluxes ($\mathbf{NJ}$), and fluxes out of the system ($\mathbf{J_{out}}$),

$$\frac{\mathrm{d}\,\mathbf{C}}{\mathrm{d}\,t} = \mathbf{J}_{\text{in}} + \mathbf{NJ} - \mathbf{J}_{\text{out}}. \tag{1}$$

Since we assumed first order kinetics, the differential equation can be written as

$$\frac{\mathrm{d}\,\mathbf{C}}{\mathrm{d}\,t} = \mathbf{J}_{\text{in}} + \begin{pmatrix} -k_9 & 0 & 0 & 0 \\ k_2 & -k_6 & 0 & 0 \\ k_3 & 0 & -k_7 & 0 \\ k_4 & 0 & 0 & -k_8 \end{pmatrix} \mathbf{C}, \tag{2}$$

with $k_9 = k_2 + k_3 + k_4 + k_5$ and $\mathbf{J}_{\text{in}}(t) = \begin{pmatrix} C_0^{\text{free\_caf}} \exp(-k_1 t) & 0 & 0 & 0 \end{pmatrix}^{\text{T}}$.

Together with some initial conditions $\mathbf{C}_0$ the system of ordinary differential equations (2) can be solved analytically. Note that due the preceding fasting period, we assumed that only the initial caffeine concentration C_0^{caf} in the sweat is zero, but not the other metabolites [6], i.e.

$$\mathbf{C}_0 = \begin{pmatrix} 0 \\ C_0^{\text{par}} \\ C_0^{\text{bro}} \\ C_0^{\text{phy}} \end{pmatrix}. \tag{3}$$

Thus, we explicitly kept the initial concentration of paraxanthine, theobromine, and theophylline as fitting parameters in the model. However, we do know the amount of ingested caffeine and thus can compute the initial (free) caffeine concentration,

$$C_0^{\text{free\_caf}} = \frac{M_{\text{dose}}}{M_{\text{donor}}} \frac{f}{v_{\text{D}}^{\text{caf}}}, \tag{4}$$

where M_{dose} is the mass of ingested caffeine, M_{donor} is the bodymass of the donor, f is the bioavailability, and $v_{\text{D}}^{\text{caf}}$ is the specific volume of distribution of caffeine. The literature value for $v_{\text{D}}^{\text{caf}}/f$ is listed in Supplementary Table 7.

With these settings the solution to (2) reads

$$c^{\text{free\_caf}} = \mathrm{e}^{-k_1 t} \tag{5}$$

$$c^{\text{caf}} = \frac{k_1}{k_9 - k_1}\left(\mathrm{e}^{-k_1 t} - \mathrm{e}^{-k_9 t}\right) \tag{6}$$

$$c^{\text{par}} = k_1 k_2 \left[F(k_1, k_6, k_9) + F(k_6, k_1, k_9) + F(k_9, k_1, k_6)\right] + c_0^{\text{par}} \mathrm{e}^{-k_6 t} \tag{7}$$

$$c^{\text{bro}} = k_1 k_3 \left[F(k_1, k_7, k_9) + F(k_7, k_1, k_9) + F(k_9, k_1, k_7)\right] + c_0^{\text{bro}} \mathrm{e}^{-k_7 t} \tag{8}$$

$$c^{\text{phy}} = k_1 k_4 \left[F(k_1, k_8, k_9) + F(k_8, k_1, k_9) + F(k_9, k_1, k_8)\right] + c_0^{\text{phy}} \mathrm{e}^{-k_8 t} \tag{9}$$

with

$$F(x, y, z) = F(x, y, z, t) = \frac{\exp(-xt)}{(x - y)(x - z)} \tag{10}$$

and

$$\mathbf{c} = \frac{\mathbf{C}}{C_0^{\text{free\_caf}}}, \quad c^{\text{free\_caf}} = \frac{C^{\text{free\_caf}}}{C_0^{\text{free\_caf}}} \tag{11}$$

where $\mathbf{c}$, c^i, and c_0^i denote corresponding normalised (and thus unit-less) metabolite concentrations.

The relation between the measured mass, $\tilde{\mathbf{M}}(t)$, and the metabolite concentration, $\mathbf{C}(t)$, is given over the sweat volume, V_{sweat}, as

$$\tilde{\mathbf{M}}(t) = \mathbf{C}(t) V_{\text{sweat}}(t), \tag{12}$$

were we implicitly made use of our central assumption that

$$V_{\text{sweat}}(t) = V_{\text{sweat}}^{\text{caf}}(t) = V_{\text{sweat}}^{\text{par}}(t) = V_{\text{sweat}}^{\text{bro}}(t) = V_{\text{sweat}}^{\text{phy}}(t). \tag{13}$$

The measured mass is also given via the calibration curve ($y = ax$, see Supplementary Table 8 and Fig. 2c in the main text), which connects it to the measured concentration, $\tilde{\mathbf{C}}(t)$, and the sample volume, V_{sample},

$$\tilde{\mathbf{M}}(t) = \mathrm{diag}(\mathbf{a}) \tilde{\mathbf{C}}(t) V_{\text{sample}}. \tag{14}$$

Here, $\mathrm{diag}(a)$ denotes an 4×4 diagonal matrix whose entries are the 4 elements of (vector of) the slopes of the calibration curves listed in Supplementary Table 8. Finally, combining Equations 11, 12, and 14 gives

$$\mathrm{diag}(\mathbf{a}) \tilde{\mathbf{C}}(t) V_{\text{sample}} \frac{M_{\text{donor}}}{M_{\text{dose}}} \frac{v_{\text{D}}^{\text{caf}}}{f} = \mathbf{c}(t) V_{\text{sweat}}(t), \tag{15}$$

where the left hand side only contains known constants or measured variables and the right hand side contains all unknown parameters. Thus, (15) can be used for fitting $V_{\text{sweat}}(t)$ as well as the kinetic constants in $\mathbf{c}(t)$.

With $\mathbf{c}(t)$ and $V_{\text{sweat}}(t)$ known from the fitting of (15), the absolute metabolite concentrations plotted in Figure 5c and f of the main manuscript is given by

$$\mathbf{C}(t) = \text{diag}(\mathbf{a})\tilde{\mathbf{C}}(t)\frac{V_{\text{sample}}}{V_{\text{sweat}}(t)} = \frac{M_{\text{dose}}}{M_{\text{donor}}}\frac{f}{v_{\text{D}}^{\text{caf}}}\mathbf{c}(t) \tag{16}$$

In literature the fractions of caffeine being degraded to paraxanthine, theobromine and theophylline are frequently reported. The fractional conversion (fc) can be calculated from the fitted kinetic constants using Equation 17. A comparison of individual fc constants from donor 1 and 2 to population averages is given in the Supplementary Table 3

$$\text{fc}_i = \frac{k_i}{k_2 + k_3 + k_4} \qquad \text{with} \;\; i \in \{2, 3, 4\} \;\; \text{for} \;\; \{\text{par, bro, phy}\} \tag{17}$$

6 Supplementary Note 4: Sensitivity analysis

6.1 Aim

In the caffeine capsule study we tried to standardize our experimental setup as much as possible. Specifically, we asked volunteers to fast foods that are known to modify the activity of caffeine processing cytochrome P450 (CYP) enzymes. However, the interpretation of the sweat metabolome is not only complicated by problems of experimental design or measurement errors but by a magnitude of intrinsic, confounding factors like the variability of the sweat volume across time courses and people or differences in precipitation between metabolites. Caffeine and its major degradation products have a similar chemical structure, and thus we assumed identical precipitation characteristics for all four of them in our model, cf. (13). Nevertheless, our mathematical model contains $11 + j$ free parameters — eight kinetic constants, three initial concentrations, and for each of the j time points one sweat volume. In the following we present our work flow and systematically investigate (i) the influence of variations in the sweat volume (ii) the influence of experimental errors (iii) the influence of different fitting methods on our model's ability to correctly identify these parameters. A list of symbols used in this section is given in Supplementary Table 6.

6.2 Methods

6.2.1 Work Flow

Similar to [7] we assessed the ability of our mathematical model to correctly fit free parameters in comparison to synthetic data. In short, concentration profiles for caffeine, paraxanthine, theobromine and theophylline were calculated according to Equations 6–9 with parameters listed in Supplementary Table 9 at $j = 20$ time points $\tau = \{0, 0.25, 0.5, 0.75, 1, 1.5, 2, 3, 4, 5, 6, 7, 8, 9, 10, 11, 12, 13, 14, 24\}$ h (unless stated otherwise). The resulting original concentration time series was subsequently artificially obstructed. In our case the obstruction was two-fold,(i) trough a randomly sampled sweat volume, and (ii) trough a randomly sampled experimental error. Next, the obstructed data ($\widetilde{\mathbf{m}}$) was used as input for data fitting and the model parameters were estimated. The fit was repeated n times for the same $\widetilde{\mathbf{m}}$ (inner Monte Carlo sampling) and the best result (namely the one with the lowest loss value) of the n replicates was stored. To get statistical significance this process was done for $i = 300$ (unless stated otherwise) replicates (i. e. the outer Monte Carlo sampling).

Since the original parameters were initially known it was subsequently possible to compare true model parameters and fitted parameters and, therefore, estimate the error associated to the data fitting procedure. The workflow was implemented with Python 3.7 and a scheme of it is shown in Supplementary Figure 12.

6.2.2 Data Creation

With assumed reasonable kinetic parameters (Supplementary Table 9) the unit-less concentrations ($\mathbf{c}$) of caffeine, paraxanthine, theobromine and theophylline were calculated using our model (Supplementary Figure 11, Equations 6 – 9) at $j = 20$ time points (unless stated otherwise). The variability of sweat flux on the finger tips spans between 0.05 and 0.62 $\mathrm{mg\,cm^{-2}\,min^{-1}}$ [8, 9]. The sweat volume, V_{sweat}, is calculated from the sweat flux by multiplying by 2 $\mathrm{cm^2}$ (sampling area) and 2 min (after washing hands 1 min of free sweating and 1 min of sweat sampling). We assumed a truncated normal distribution of the sweat volume, V_{sweat}, with the mean of both literature values ($\mu_{V_{\mathrm{sweat}}} = 1.34\,\mathrm{\mu L}$) and the standard deviation of both values halved ($\sigma_{V_{\mathrm{sweat}}} = 0.57\,\mathrm{\mu L}$).

$$V_{\mathrm{sweat}} \sim \mathcal{N}(1.34\,\mathrm{\mu L}, 0.57^2\,\mathrm{\mu L}) \quad \text{with} \quad 0 < V_{\mathrm{sweat}} < \infty. \tag{18}$$

In Supplementary Figure 13b the probability density of the V_{sweat} values of all simulations is shown. 96.4 % of them are within literature bounds.

Next, the experimental error was sampled. Unless stated otherwise, we assumed a normal error distribution with $\mu_\epsilon = 1$ and $\sigma_\epsilon = 10\%$ for the experimental error, $\boldsymbol{\epsilon}$,

$$\boldsymbol{\epsilon} \sim \mathcal{N}(1, 0.1^2) \quad \text{with} \quad 0 < \boldsymbol{\epsilon} < \infty. \tag{19}$$

We point out that unlike V_{sweat}, $\boldsymbol{\epsilon}$ is not the same for every metabolite at one time point, t, and is, therefore, a vector. Moreover, the measurements of the caffeine capsule study were performed in technical replicates, and thus $\boldsymbol{\epsilon}$ is sampled twice for every time point. The probability density of $\boldsymbol{\epsilon}$ is shown in Supplementary Figure 13d. Both, V_{sweat} and $\boldsymbol{\epsilon}$ are applied to the theoretical concentrations as multiplication,

$$\tilde{\mathbf{m}} = \text{diag}(\mathbf{c})\ V_{\text{sweat}}\ \boldsymbol{\epsilon}. \tag{20}$$

As an example, the steps of data processing are visualized in Supplementary Figure 13. Supplementary Figure 13a shows, the size of the sampled V_{sweat}s at their respective time points and Supplementary Figure 13b shows the true concentration (c) as black line and the artificially errored data points as blue dots or orange crosses (0 % and 10 % error, respectively). For simplicity reasons we do not show the final data points ($\tilde{\mathbf{m}}$) which additionally includes the sweat volume and the remaining three metabolites.

6.2.3 Fitting Procedure

The data fitting procedure was implemented in Python using SciPy's `curve_fit` function [10]. As minimization method the exact trust region reflective (`'trf'`) algorithm was selected. Numerical tolerance settings were kept at default (10^{-8}). The general, adaptive, and robust loss [11] required two more fitting parameters which were optimized in a separate minimization problem inside of the loss function using SCiPy's `minimize` and the truncated Newton (`'TNC'`) algorithm. The numerical tolerance was set to 10^{-10} being more accurate than the main minimization problem. Moreover, each input data set was fitted n times as Monte Carlo (MC) replicates. The fit with the lowest overall loss was chosen as the best option and saved for subsequent analysis. The bounds for the model parameters are listed in Supplementary Table 9. All values reported in literature are well within these bounds [12, 13, 1, 2, 14, 15, 16, 17]. Nevertheless, many hyperparameters still needed to be manually tuned and in this study we focused on finding optimal settings for (i) the choice of loss function,(ii) the number of Monte Carlo replicates, and (iii) the number of time points.

6.2.4 Data Analysis

Two quality parameters of the fit were calculated: (i) the coefficient of variation (CV in %) giving the relative width of the fitted parameter distribution around the true value (μ, Equation 21) and (ii) the median of the relative error (MRE in %) which gives the offset of the fitted parameter distribution to the true value (Equation 22).

$$\text{CV} = \sqrt{\frac{1}{i}\sum_{m=1}^{i}\left(\frac{x_m - \mu}{\mu}\right)^2} \tag{21}$$

$$\text{MRE} = \text{median}\left(\frac{x_m - \mu}{\mu}\right) \tag{22}$$

$$\text{for} \quad x_m (m = 1, ..., i)$$

Generally speaking CV is a measurement of precision, whereas MRE is a measurement of accuracy. In rare cases some extreme outliers were impacting the calculation of CV of V_{sweat}.

This happened when true V_{sweat} values were sampled outside of the bounds of the fit (close to 0 µL). To get better estimates of CV, all outliers with more than 1000% deviation from the true value were excluded. However, the number of excluded outliers never exceeded 0.1% of the whole sample set for all simulations except one (a comparison simulation without V_{sweat} normalisation, Supplementary Table 17). To give an overall estimate of a certain fitting procedure's performance, the average of the absolute MREs and CVs of all model parameters was calculated. Error distributions were plotted as histograms with 21 bins. To test for statistical significance of error distribution we used Levene's test for the similarity of (error) variances [18]. Raw data and analysis code is available at GitHub (`https://github.com/Gotsmy/finger_sweat`)[19].

6.3 Results

6.3.1 Loss Functions

Three losses (Cauchy, Huber, and Soft-l1) which are part of the SciPy package [10] and implemented in its `curve_fit` function and a forth, general, adaptive, and robust loss described by Barron 2019 [11] (from now on referred to as Robust loss) were tested with $n = 100$ inner and $i = 300$ outer MC replicates. Additionally, we analysed differences in the performance with respect to accuracy when the loss was calculated with absolute errors or the maximum of either absolute or relative errors for every time point. The performances of the loss functions are shown in Supplementary Figure 14. For most cases, we found that estimating the loss from the maximum of absolute and relative error resulted in better (*i.e.* smaller) CVs and MREs than just taking the absolute errors into account. Moreover, the Max Robust loss was clearly performing best, followed by Max Cauchy and all others being more inaccurate. Therefore, we continued to use the Robust loss function calculated from the maximum of absolute and relative error (max Robust) in all subsequent simulations.

6.3.2 Inner Monte Carlo Replicates

As mentioned in the Section 6.2.3, MC replicates were performed for every time course data set, and the result with the best loss was selected. This was done to sample the whole solution space in an unbiased manner and to prevent the estimation of incorrect fitting parameters from convergence in local minima. Simulations (with Max Robust loss and $i = 300$ outer MC replicates) for different numbers of inner MC replicates, n, were performed. The results are shown in Supplementary Figure 15. Generally, there are big improvements of CV and MRE between $n = 1$ and $n = 100$. Less improvements are visible with more inner MC replicates. Therefore, and because of the additional computational load with increasing n we decided to use 100 inner MC replicates for the data fitting procedure and all subsequent simulations.

6.3.3 Sampling Time Points

Next, we considered different amounts of sampling time points and how they affect the error associated to data fitting. The sampling time points used in the simulations closely resemble the time points used for finger sweat data acquisition in the caffeine capsule study (Supplementary Table 12). In that study we found a high variability of late concentrations that were fitted. Thus we were interested whether it is advisable to not use the late values at all for the fitting procedure. The number of time points for which data was created was defined as j and simulations were performed with $n = 100$ inner and $i = 300$ outer MC replicates using the max Robust loss. An overview of the time points is shown in Supplementary Table 12. The change of the CV and MRE with different time points is plotted in Supplementary Figure 16. Taking more time points into account improves the CV for the time-independent kinetic constants and initial parameters. In contrast the CV across all time points of time-dependent $V_{\text{sweat}}(t)$ and $\mathbf{c}(t)$ stays constant or even worsens slightly for larger j. This indicates that the error variance of

time-dependent parameters is not constant over time (as further investigated in Section 6.3.4). Interestingly, the number of sampling time points does not have a strong impact on MRE with some parameters improving and some deteriorating in a seemingly random pattern. One peculiarity was k_5 (constant of caffeine degradation) which was the most overestimated parameter with $j = 11$ and then went on to be most underestimated parameter with $j = 20$.

6.3.4 Time Dependence

Previous results indicated that, when including time points many hours after the initial ingestion of the caffeine capsule, the CV of time-dependent parameters worsened. Therefore, we calculated the distribution of relative error for $V_{\text{sweat}}(t)$ and $\mathbf{c}(t)$ on each time point of a simulation. All distributions were taken from one simulation with $n = 100$ inner MC replicates, $i = 3000$ outer MC replicates and $j = 20$ time points using the max Robust loss.

The distribution of relative errors of the V_{sweat} at different time points is shown in Supplementary Figure 17a. Interestingly, the distribution for early time points ($0\,\text{h} \leq t \leq 1\,\text{h}$) was comparably wide (CV roughly > 20 %). For time points in the middle of the curve ($1.5\,\text{h} \leq t \leq 11\,\text{h}$) CV was the lowest ($10\,\% < \text{CV} < 20\,\%$). Thereafter, the more time there was between sampling and caffeine intake, the worse the V_{sweat} predictions got (*i. e.* wider error distribution, CV > 20 %). Finally, there is a clearly visible drop in precision and accuracy for $t = 24$ h (black line, CV > 30 %, $|\text{MRE}| > 5$ %).

Similarly to V_{sweat}, a time-dependent change in error distribution is visible for unit-less metabolite concentrations as shown in Supplementary Figure 17b–e. Concretely, we observed higher CVs in early samples ($0\,\text{h} \leq t \leq 1\,\text{h}$), the smallest errors in the middle section ($1.5\,\text{h} \leq t \leq 6\,\text{h}$) and an increase in CV thereafter. Generally, the CVs for concentrations were smaller than for V_{sweat}, however, time point $t = 24\,\text{h}$ clearly performed worst for all parameters (black lines, CV $> 30\%$).

Early measurements of the caffeine capsule study were done with fewer sampling time points (simulation E15, Supplementary Table 12). To estimate the CV and MRE with this set-up a simulation with $n = 100$, $i = 3000$, and the max Robust loss was performed and the time-dependent results that were used to calculate confidence intervals of Supplementary Table 2 are shown in Supplementary Table 15.

6.3.5 Experimental Error

So far a fixed experimental error of 10% was assumed. In this section we investigate the influence of experimental error on the size of CV and MRE. The changes in size of fitting errors are shown in Supplementary Figure 18 for experimental errors between $\sigma_\epsilon = 1\%$ and 30% ($i = 300$, $n = 100$, max Robust loss). As expected, as the experimental errors propagated through the fitting procedure and lead to increased CVs and MREs. Interestingly, the propagation follows a linear relationship, however, the slopes of the increase of CVs were different for different parameters. Constants (full and dotted lines) seem to be impacted more than the time-dependent concentrations (dashed/dotted lines). Only the time-dependent V_{sweat} (dashed line) increased at a similar rate as the time-independent parameters.

6.3.6 V_{sweat} Normalisation

To analyse the impact of the V_{sweat} normalisation, we compared two fitting procedures. First we used the procedure as described in the previous section. Second we assumed a constant sweat volume $\mu_{V_{\text{sweat}}} = 1.34\,\mu\text{L}$ (Equation 18) across all time points (being equivalent to no V_{sweat} normalisation). Simulations were done with $i = 3000$ inner and $n = 100$ outer MC replicates and max Robust loss. The distribution of fitting errors around the true value for the kinetic constants of the model are shown in Supplementary Figure 19. MREs are found to be close to the

true value in both cases. However, the distribution of errors, CV, is clearly narrower with V_{sweat} normalisation than without (Supplementary Table 17). On average the V_{sweat} normalisation improves the CV of fitting constants by 19%. The highest differences in CV are found for V_{sweat} itself (79%), followed by the kinetic constants k_i (25%) and the smallest differences for the concentrations (4.5%). Generally, the results showed that with V_{sweat} normalisation the error variances for all model parameters were significantly smaller than without V_{sweat} normalisation (Levene's test, all P-values $< 10^{-10}$, sample size for time-independent constants $= i$, for $V_{\text{sweat}}, c^{\text{par}}, c^{\text{bro}}$, and $c^{\text{phy}} = i \cdot j$, and for $c^{\text{caf}} = i \cdot (j-1)$).

6.4 Discussion

Here we investigated the error associated to a fitting procedure that accounts for variable sweat volume includes an V_{sweat}. We found that the best loss function (i.e. the loss function with the lowest CV and MRE) for this task is the general, adaptive, and robust loss [11]. Our results showed that using the maximum of absolute and relative error per time point for calculation of the loss gives better accuracy compared to just the absolute error. Next, we investigated the optimal number of innter Monte Carlo replicates. The idea behind taking many MC replicates is to avoid getting stuck in local minima. We found that up to 100 MC replicates the quality parameters of the fit improved. With more than 100 MC replicates, however, no substantial improvement of the fit's accuracy and precision was observed. Therefore, and for the reason of reducing computational expense, we chose 100 MC replicates for the data fitting procedure.

Error estimation for simulations with different number of sampling points clearly showed an advantage of increasing the number of samples. Thus, we concluded that even samples measured after more than 24 h after caffeine capsule ingestion can improve the precision of time-independent fitting parameters (compare $j = 19$ and $j = 20$ of Supplementary Figure 16a). However, this lead to an overall decrease of quality of estimation of time-dependent parameters. This decline, however, was mainly due to high errors in late concentration and V_{sweat} estimates and, therefore, they should be treated very conservatively. This effect makes sense since so late the concentrations are relatively low and small absolute deviations can have big relative errors as consequence.

Additionally, we showed that the fitting error linearly increased with the assumed experimental error, which indicates that the method is robust, even to bigger disturbances of the input data. However, we argue that expecting an experimental error of 10% is reasonable since variations within one donor are expected to be small and potential technical variations are corrected for by division with the internal standard.

Finally, we compared the CV and MRE for a fitting procedure with and without V_{sweat} normalisation and we demonstrated that indeed explicitly using V_{sweat} values in the model significantly improves the precision of the fit.

7 Supplementary References

[1] Bonati, M. *et al.* Caffeine disposition after oral doses. *Clin. Pharmacol. Ther.* **32**, 98–106 (1982).

[2] Lelo, A., Birkett, D., Robson, R. & Miners, J. Comparative pharmacokinetics of caffeine and its primary demethylated metabolites paraxanthine, theobromine and theophylline in man. *Brit. J. Clin. Pharmaco.* **22**, 177–182 (1986).

[3] Kamimori, G. H. *et al.* The rate of absorption and relative bioavailability of caffeine administered in chewing gum versus capsules to normal healthy volunteers. *Int. J. Pharmaceut.* **234**, 159–167 (2002).

[4] Skupinska, K., Misiewicz, I. & Kasprzycka-Guttman, T. A comparison of the concentration–effect relationships of PAHs on CYP1A induction in HepG2 and mcf7 cells. *Arch. Toxicol.* **81**, 183–200 (2006).

[5] Sonner, Z. *et al.* The microfluidics of the eccrine sweat gland, including biomarker partitioning, transport, and biosensing implications. *Biomicrofluidics* **9**, 031301 (2015).

[6] Denaro, C. P., Brown, C. R., Wilson, M., Jacob III, P. & Benowitz, N. L. Dose-dependency of caffeine metabolism with repeated dosing. *Clin Pharmacol Ther* **48**, 277–285 (1990).

[7] Széliová, D. *et al.* Error propagation in constraint-based modeling of Chinese hamster ovary cells. *Biotechnol. J.* **16**, 2000320 (2021).

[8] Taylor, N. A. & Machado-Moreira, C. A. Regional variations in transepidermal water loss, eccrine sweat gland density, sweat secretion rates and electrolyte composition in resting and exercising humans. *Extrem Physiol. Med.* **2**, 4 (2013).

[9] Ando, H. & Noguchi, R. Dependence of palmar sweating response and central nervous system activity on the frequency of whole-body vibration. *Scand. J. Work Environ. Health* **29**, 216–219 (2003).

[10] Virtanen, P. *et al.* SciPy 1.0: Fundamental algorithms for scientific computing in python. *Nat. Methods* **17**, 261–272 (2020).

[11] Barron, J. T. A general and adaptive robust loss function. In *2019 IEEE/CVF Conference on Computer Vision and Pattern Recognition (CVPR)*, 4331–4339 (IEEE, 2019). URL `https://doi.org/10/gg4wn3`.

[12] Grzegorzewski, J. *et al.* PK-DB: Pharmacokinetics database for individualized and stratified computational modeling. *Nucleic Acids Res.* **49**, D1358–D1364 (2020).

[13] Arnaud, M. & Welsch, C. Theophylline and caffeine metabolism in man. In *Theophylline and other Methylxanthines / Theophyllin und andere Methylxanthine*, 135–148 (Vieweg+Teubner Verlag, 1982). URL `https://doi.org/10/gkghqt`.

[14] Holstege, A., Staiger, M., Haag, K. & Gerok, W. Correlation of caffeine elimination and child's classification in liver cirrhosis. *Klin. Wochenschr.* **67**, 6–15 (1989).

[15] Jeppesen, U., Loft, S., Poulsen, H. E. & Brosen, K. A fluvoxamine-caffeine interaction study. *Pharmacogenetics* **6**, 213–222 (1996).

[16] Akinyinka, O., Sowunmi, A., Honeywell, R. & Renwick, A. The effects of acute falciparum malaria on the disposition of caffeine and the comparison of saliva and plasma-derived pharmacokinetic parameters in adult Nigerians. *Eur. J. Clin. Pharmacol.* **56**, 159–1065 (2000).

[17] Laizure, S. C. *et al.* Comparison of caffeine disposition following administration by oral solution (energy drink) and inspired powder (AeroShot) in human subjects. *Br. J. Clin. Pharmacol.* **83**, 2687–2694 (2017).

[18] Levene, H. Robust tests for equality of variances. *Contributions to probability and statistics. Essays in honor of Harold Hotelling* 279–292 (1961).

[19] Brunmair, J. *et al.* Finger sweat analysis enables short interval metabolic biomonitoring in humans. *GitHub*, doi: 10.5281/zenodo.5222967 (2021).
