# Supplementary material for: Finger sweat analysis enables short interval metabolic biomonitoring in humans
Source: Nat Commun. 2021 Oct 13;12:5993. doi: 10.1038/s41467-021-26245-4 (PMC8514494; doi:10.1038/s41467-021-26245-4)
Supplement: Supplementary file 3 — Description of additional supplementary files [file 41467_2021_26245_MOESM3_ESM.docx]

Supplementary Data 1: **List of molecules identified by the Compound Discoverer Software and transferred to the Tracefinder Software (both Thermo Fisher Scientific) for the positive ion mode.**

Supplementary Data 2: **List of all molecules from the coffee extract identified by the Compound Discoverer Software.** Highlighted in grey are molecules that could also be detected in finger sweat samples.
