# Supplementary material for: Finger sweat analysis enables short interval metabolic biomonitoring in humans
Source: Nat Commun. 2021 Oct 13;12:5993. doi: 10.1038/s41467-021-26245-4 (PMC8514494; doi:10.1038/s41467-021-26245-4)
Supplement: Supplementary file 6 — Reporting Summary [file 41467_2021_26245_MOESM6_ESM.pdf]

nature research

Corresponding author(s): Christopher Gerner

Last updated by author(s): Aug 13, 2021

Reporting Summary

Nature Research wishes to improve the reproducibility of the work that we publish. This form provides structure for consistency and transparency in reporting. For further information on Nature Research policies, see our Editorial Policies and the Editorial Policy Checklist.

Statistics

For all statistical analyses, confirm that the following items are present in the figure legend, table legend, main text, or Methods section.

n/a	Confirmed	
☐	☒	The exact sample size (*n*) for each experimental group/condition, given as a discrete number and unit of measurement
☐	☒	A statement on whether measurements were taken from distinct samples or whether the same sample was measured repeatedly
☐	☒	The statistical test(s) used AND whether they are one- or two-sided<br>*Only common tests should be described solely by name; describe more complex techniques in the Methods section.*
☒	☐	A description of all covariates tested
☐	☒	A description of any assumptions or corrections, such as tests of normality and adjustment for multiple comparisons
☐	☒	A full description of the statistical parameters including central tendency (e.g. means) or other basic estimates (e.g. regression coefficient) AND variation (e.g. standard deviation) or associated estimates of uncertainty (e.g. confidence intervals)
☐	☒	For null hypothesis testing, the test statistic (e.g. *F*, *t*, *r*) with confidence intervals, effect sizes, degrees of freedom and *P* value noted<br>*Give P values as exact values whenever suitable.*
☒	☐	For Bayesian analysis, information on the choice of priors and Markov chain Monte Carlo settings
☒	☐	For hierarchical and complex designs, identification of the appropriate level for tests and full reporting of outcomes
☐	☒	Estimates of effect sizes (e.g. Cohen's *d*, Pearson's *r*), indicating how they were calculated

Our web collection on statistics for biologists contains articles on many of the points above.

Software and code

Policy information about availability of computer code

Data collection	Raw data was generated with the Q Exactive HF coupled to a Vanquish UHPLC system (both Thermo Fisher Scientific), which were controlled with Xcalibur 4.0.
Data analysis	The Compound Discoverer Software 3.1 and the Tracefinder Software 4.1 (Thermo Fisher Scientific) have been applied to identify molecules in sweat and automatically obtain peak areas from identified compounds. Microsoft Excel (version 1808) was used to further process data for statistical analysis. Microsoft PowerPoint (version 1808), Smart Sevier (www.servier.com), Thermo Fisher Scientific's Freestyle (version 1.3.115.19) and Biorender (www.biorender.com) was used for creating figures. GraphPad Prism (version 6.07) was used for normality tests (D'Agostino-Pearson tests as well as Kolmogorov-Smirnov tests with Dallal-Wilkinson-Lilliefors p-value) as well as for tow-tailed, paired t-tests or Wilcoxon Signed Rank Tests. Vulcano plots were obtained using Perseus Software (version 1.6.12.0). Shared-control estimation plots were generated with https://www.estimationstats.com/. PCA of kinetic parameters (Figure 5d) was performed with Python 3.7 and scikit-learn (version 0.23.2). Levene-test in sensitivity analysis was performed with Python 3.7 and scipy (version 1.6.1). The mathematical modelling and sensitivity analysis was performed with Python 3.7 heavily relying on packages scipy (version 1.6.1) and robust-loss-pytorch (version 0.0.2 https://github.com/jonbarron/robust_loss_pytorch@9831f1db8006105fe7a383312fba0e8bd975e7f6). Detailed code can be found at https://github.com/Gotsmy/finger_sweat or rather http://doi.org/10.5281/zenodo.5222967)

For manuscripts utilizing custom algorithms or software that are central to the research but not yet described in published literature, software must be made available to editors and reviewers. We strongly encourage code deposition in a community repository (e.g. GitHub). See the Nature Research guidelines for submitting code & software for further information.

Data

Policy information about availability of data

All manuscripts must include a data availability statement. This statement should provide the following information, where applicable:

- Accession codes, unique identifiers, or web links for publicly available datasets
- A list of figures that have associated raw data
- A description of any restrictions on data availability

The datasets that support the findings of this this study have been deposited in the MetaboLights repository with the accession numbers MTBLS2772 (https://www.ebi.ac.uk/metabolights/MTBLS2772) and MTBLS2776 (https://www.ebi.ac.uk/metabolights/MTBLS2776). This data contains everything used to create the corresponding figures. Source data are provided with the manuscript as excel sheet.

Field-specific reporting

Please select the one below that is the best fit for your research. If you are not sure, read the appropriate sections before making your selection.

- [x] Life sciences
- [ ] Behavioural & social sciences
- [ ] Ecological, evolutionary & environmental sciences

For a reference copy of the document with all sections, see nature.com/documents/nr-reporting-summary-flat.pdf

Life sciences study design

All studies must disclose on these points even when the disclosure is negative.

Sample size	Here we report explorative studies rather than studies proving a hypothetic effect of a drug or similar intervention. Thus, we did not perform calculation of sample sizes. We chose the sample size of 47 donors which proved to be sufficient to obtain effect size plots providing information regarding pharmacokinetics and to observe significant endogenous responses.
Data exclusions	No data was excluded from the analyses.
Replication	The technical reproducibility was demonstrated by the determination of calibration curves (Figure 2) and the coefficient of variation of the internal standard. The reproducibity of individual sweat sample analyses was illustrated by the principal component analysis shown in Figure 1. The reproducibility of caffeine metabolic product formation in different individuals was demonstrated in the effect size plot (Figure 4). The reproducibility of individual metabolic properties was demonstrated in the PCA plot shown in Figure 5. Here, seven of the 40 participants were repeatedly analyzed. The visible cluster of repeated determinations of these individuals demonstrates that the k-values corresponding to metabolic properties of the same individiuum are reproducible. In addition, regarding the in vitro experiment with HepG2 cells, three independet replicates with technical duplicates demonstrate high reproducibility supporting the conclusions.
Randomization	We are presenting feasibility studies introducing a new methodology rather than results of clinical studies based on established methods. This is why we have not used randomization yet. As we performed comparative analyses mainly based on time course analyses rather than group comparisons, randomization was not applicable.
Blinding	Blinding was not applied due to reasons explained above. However, the time course analysis data of control individuals in comparison to consumers are shown in Supplementary Figure 2. Indeed, the groups can be unambiguously discerned based on the analysis results. All analyses were performed following our lab management system. This means, each sample was encoded during measurement, decoding only occurred after finalisation of measurements.

Reporting for specific materials, systems and methods

We require information from authors about some types of materials, experimental systems and methods used in many studies. Here, indicate whether each material, system or method listed is relevant to your study. If you are not sure if a list item applies to your research, read the appropriate section before selecting a response.

Materials & experimental systems

n/a	Involved in the study	
x		Antibodies
	x	Eukaryotic cell lines
x		Palaeontology and archaeology
x		Animals and other organisms
	x	Human research participants
x		Clinical data
x		Dual use research of concern

Methods

n/a	Involved in the study	
x		ChIP-seq
x		Flow cytometry
x		MRI-based neuroimaging

Eukaryotic cell lines

Policy information about cell lines

Cell line source(s)	HepG2 cells (HB-8065) were purchased from ATCC.
Authentication	HepG2 cells were cultivated from an original ATCC vial. ATCC routine authentication procedures were performed by the company. According to the company no mycoplasma contamination was detected. Expression makers used were insulin and insulin-like growth factor II (IGF II). STR profiling was performed with Amelogenin: X,Y; CSF1P0: 10,11, D13S317: 9,13, D16S539: 12,13, D5S818: 11,12, D7S820: 10, THO1: 9, TPOX: 8,9, and vWA: 17.
Mycoplasma contamination	HepG2 cells were cultivated from an original ATCC vial. According to the product sheet no mycoplasma contamination was detected.
Commonly misidentified lines (See ICLAC register)	No commonly misidentified lines were used in this study.

Human research participants

Policy information about studies involving human research participants

Population characteristics	21 males and 19 females with ages between 20-55 years and a BMI of 21 +/- 8 were enrolled in this study. There was no exclusion criteria other than being over the age of 18. Healthy participants were enrolled.
Recruitment	Participants were recruited via advertisement at the Department of Analytical Chemistry, Faculty of Chemistry, University of Vienna. Assignment of individuals to groups (control vs coffee) occured by chance. In case of the caffeine capsule intervention study, all participants followed exactly the same protocol, thus, only one group was defined, ruling out any possible selection bias.
Ethics oversight	The study protocol was performed in accordance with the University of Vienna and has been approved by the ethical committee of the University of Vienna (reference number 00337). Written informed consent has been obtained from all donors participating in this study.

Note that full information on the approval of the study protocol must also be provided in the manuscript.
